# The intrinsically disordered SARS-CoV-2 nucleoprotein in dynamic complex with its viral partner nsp3a

**DOI:** 10.1126/sciadv.abm4034

**Published:** 2022-01-19

**Authors:** Luiza Mamigonian Bessa, Serafima Guseva, Aldo R. Camacho-Zarco, Nicola Salvi, Damien Maurin, Laura Mariño Perez, Maiia Botova, Anas Malki, Max Nanao, Malene Ringkjøbing Jensen, Rob W. H. Ruigrok, Martin Blackledge

**Affiliations:** 1 Université Grenoble Alpes, CNRS, CEA, IBS, F-38000 Grenoble, France.; 2 Structural Biology Group, European Synchrotron Radiation Facility, F-38000 Grenoble, France.

## Abstract

The processes of genome replication and transcription of SARS-CoV-2 represent important targets for viral inhibition. Betacoronaviral nucleoprotein (N) is a highly dynamic cofactor of the replication-transcription complex (RTC), whose function depends on an essential interaction with the amino-terminal ubiquitin-like domain of nsp3 (Ubl1). Here, we describe this complex (dissociation constant - 30 to 200 nM) at atomic resolution. The interaction implicates two linear motifs in the intrinsically disordered linker domain (N3), a hydrophobic helix (^219^LALLLLDRLNQL^230^) and a disordered polar strand (^243^GQTVTKKSAAEAS^255^), that mutually engage to form a bipartite interaction, folding N3 around Ubl1. This results in substantial collapse in the dimensions of dimeric N, forming a highly compact molecular chaperone, that regulates binding to RNA, suggesting a key role of nsp3 in the association of N to the RTC. The identification of distinct linear motifs that mediate an important interaction between essential viral factors provides future targets for development of innovative strategies against COVID-19.

## Teaser

Intrinsically disordered SARS-CoV-2 nucleoprotein folds around its viral partner nsp3a to form a dynamic and compact complex.

## INTRODUCTION

Severe acute respiratory syndrome coronavirus 2 (SARS-CoV-2), the origin of the respiratory coronavirus disease 2019 (COVID-19) pandemic, is a member of the betacoronavirus genus, also including Middle East respiratory syndrome (MERS)–CoV and SARS-CoV. In response to the rapid spread of this virus, it has become urgent to develop viral inhibitors that can reduce or eradicate symptoms. The processes of replication and transcription of viral RNA represent important targets for viral inhibition, and the development of rational strategies to achieve this end requires a molecular understanding of the viral replication cycle.

Coronaviridae are enveloped positive-sense single-strand RNA viruses that express their own replication machinery. Replication of the SARS-CoV-2 genome is carried out by the RNA-dependent RNA polymerase complex, whose functional modes have been investigated by cryo–electron microscopy (*1*–*3*). Betacoronaviral replication-transcription complexes (RTCs) are associated with membrane networks in the form of viral replication organelles called double-membrane vesicles (DMVs) constituted from membranes participating in the host secretory pathway (*4*). DMVs have been shown to constitute active sites of viral RNA synthesis (*5*, *6*).

The nucleoprotein of SARS-CoV-2 (N) is an essential cofactor of the replication machinery (*7*, *8*), encapsidating the viral genome, providing protection from the host cell environment, and playing an essential role in regulating gene transcription (*9*). N is produced at high levels in infected cells, making it an important marker for infection, and has also been linked to the perturbation of numerous host processes (*8*). Understanding the conformational behavior and function of N is therefore important for both potential therapeutic and diagnostic treatment of COVID-19 (*10*) as well as vaccine development (*11*). N colocalizes to the RTC in betacoronaviruses (*12*–*14*), although the molecular basis of its role in regulation of replication and transcription remains poorly understood. It has been shown in mouse hepatitis virus (MHV) that this colocalization depends on an essential interaction with the N-terminal component of nonstructural protein 3 (nsp3) (*15*, *16*).

**Fig. 1.**
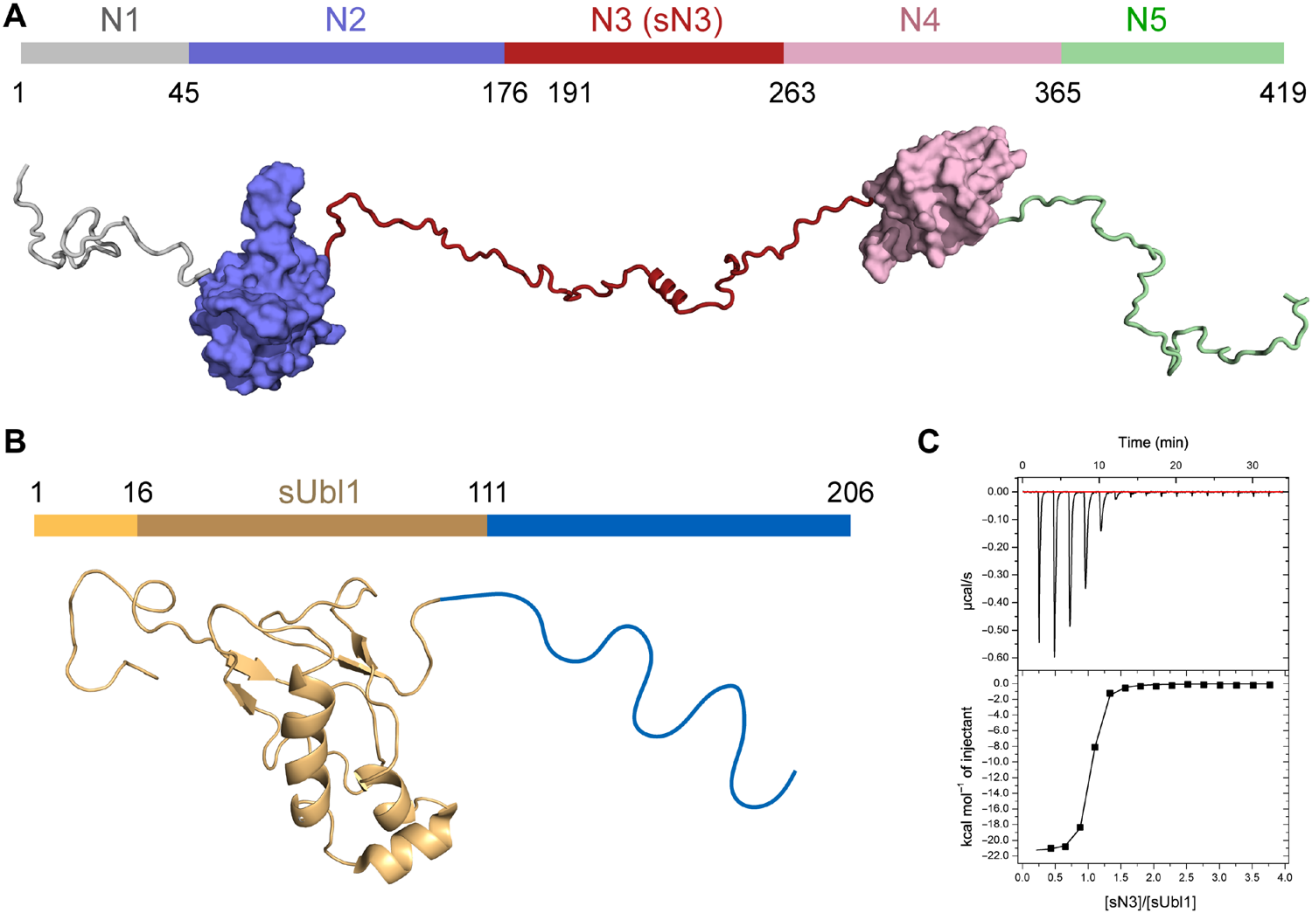
Figurative representation of N and nsp3a SARS-CoV-2 proteins. (**A**) Domain representation of N. Domain N1 is intrinsically disordered. Domain N2 [otherwise known as N-terminal domain (NTD) or RNA binding domain], whose recently determined x-ray crystallographic structure is shown here [PDB code 6m3m (*23*)]. Domain N3 is also intrinsically disordered, comprising a serine-arginine (SR)–rich region, followed by a short helix. Domain N4 [also known as the C-terminal domain (CTD] is responsible for dimerization of N [structure shown is taken from the crystal structure (*28*), PDB code 3wzo]. The basic domain N5 is also intrinsically disordered. (**B**) Nsp3a. The N-terminal domain of nsp3a has a ubiquitin-like fold [the NMR structure of nsp3a of SARS-CoV (PDB code 2idy) is shown here (*49*)]. The C-terminal domain of nsp3a is intrinsically disordered and strongly acidic. (**C**) ITC of sUbl1 (16 to 111) in complex with sN3 (191 to 263) (235F or alpha variant). These domains represent the necessary domains for interaction between N and nsp3a (fig. S1 and table S1).

Coronaviral N is highly dynamic, and three of the five domains (numbered N1 to N5 here; see Fig. 1A) are intrinsically disordered regions (IDRs), intercalated with two folded domains [N2 and N4, also known as N- and C-terminal domains (NTD and CTD)] (*17*). The structural features of betacoronaviral N are strongly conserved, with 90% sequence identity between SARS-CoV and SARS-CoV-2. N is dimeric and highly basic, and RNA binding is thought to be cooperative and to involve all five domains of N from SARS-CoV, where it plays a role in both chaperoning and RNA packaging (*8*, *18*). Electron tomography identified numerous N-RNA complexes within SARS-CoV-2 virions, apparently associated with different segments of the 35-kb genomes (*19*, *20*). The N3 IDR (175 to 263) comprises a serine-arginine (SR)–rich domain that is phosphorylated in infected cells, a modification that plays a role in both function and localization of SARS-CoV N (*21*).

The folded domains N2 and N4 are not thought to interact with each other in SARS-CoV (*17*). N4 is responsible for homodimerization (*17*) and, together with N5, is thought to contribute to the formation of higher-order assemblies that constitute the nucleocapsid (*22*). N2 has been investigated in isolation by x-ray crystallography (*23*, *24*) and nuclear magnetic resonance (NMR) spectroscopy (*25*), and its interaction with RNA has been investigated by NMR (*25*–*27*). The structure of N4 has been determined by x-ray crystallography (*24*, *28*), and N was shown to form dimers or higher-order oligomers under different experimental conditions (*28*–*30*). Molecular modeling and single-molecule fluorescence resonance energy transfer (*31*) were used to probe the dynamic nature of N. The disordered character of domains N1 and N3 was verified using NMR spectroscopy (*32*, *33*), revealing the presence of a central helical element in N3 and the flexible nature of both domains in the presence of N2. A recent study also characterized the isolated SR region of N3 in its phosphorylated and nonphosphorylated forms (*34*). Mass spectrometry was used to identify a number of autocatalytic sites in N, two of which are present in N3 (*30*). N2 and full-length N have been shown to bind to different RNAs (*25*, *35*, *36*).

N from SARS-CoV-2 has been shown to undergo liquid-liquid phase separation (LLPS), forming biomolecular condensates upon mixing with RNA (*31*, *34*, *35*, *37*–*41*). LLPS, mediated by dynamic interactions involving IDRs or RNA, offers an efficient mechanism to spatially and temporally control biochemical processes in the cell (*42*). In negative-sense single-stranded RNA viruses, such as rabies (*43*) and measles (*44*), components involved in viral replication have been shown to form functional membraneless condensates. In positive-sense RNA coronaviruses, there is currently no direct evidence that LLPS is related to either replication or the formation of DMVs, although it has been shown that SARS-CoV-2 RNA polymerase can localize to N:RNA droplets (*34*). Phosphorylation of N3 in the SR region was also shown to modulate the biophysical properties of these droplets (*35*, *39*–*41*).

The N cofactor nsp3 is the largest protein encoded in the SARS-CoV-2 genome, comprising 1945 amino acids, constituting up to 16 distinct domains (*45*), and playing an essential role in replication and transcription (*46*). The amino-terminal domains of nsp3, comprising a ubiquitin-like domain (Ubl1) and a highly acidic IDR, together constitute nsp3a (Fig. 1B) (*47*). Ubl1 was implicated in binding to the SR region of N in MHV (*48*), while a yeast two-hybrid approach implicated a hydrophobic linear motif slightly upstream of the SR region (*16*).

The three-dimensional (3D) structures of Ubl1 from SARS-CoV (*49*) and MHV (*50*) have been determined by NMR. Yeast two-hybrid and glutathione *S*-transferase (GST) pull-down experiments identified viral proteins nsp8 and nsp9—components of the RTC—and different domains of nsp3 as interaction partners (*51*). Notably, deletion of Ubl1 results in abrogation of viral replication (*15*) in MHV, while the conserved highly acidic IDR was dispensable for replication. The interaction between nsp3a and N from MHV was investigated using NMR (*50*), revealing apparent cooperative binding of N2 and N3, and a possible inhibitory role of N1. Nsp3a was also shown to bind single-stranded RNA in SARS-CoV (*49*), and interaction between the transcription regulatory sequence RNA (*52*) and N was suggested to inhibit the N:nsp3a interaction (*50*). Ubl1 from SARS-CoV-2 was recently found to partition into liquid droplets comprising N and RNA (*39*). X-ray crystallography revealed that the 3D structure of Ubl1 in SARS-CoV-2 [Protein Data Bank (PDB) code 7kag] is very similar to that found in SARS-CoV and MHV, while NMR shows that the structure is retained in solution and that the acidic domain is disordered (*53*).

Nsp3 is one of the nonstructural viral proteins that are essential for formation of DMVs in MERS-CoV (*54*), and is thought to play a role in trafficking N to the RTC, colocalizing with N in the vicinity of DMVs (*12*). Recent investigation of DMVs using cellular cryo–electron tomography (*4*) identified molecular pores that allow the import and export of the replication/transcription substrate and product, further highlighting the importance of this interaction. The pores are shown to be constituted of nsp3 oligomers, with nsp3a protruding into the cytosol in the vicinity of putative N oligomers, suggesting that interaction with N may regulate RNA exit and encapsidation (*4*).

Here, we use solution-state NMR, combined with isothermal titration calorimetry (ITC) and small-angle x-ray scattering (SAXS), to describe the interaction of N with nsp3a from SARS-CoV-2. We show that the interaction is mediated uniquely by the disordered N3 domain, which folds on the surface of Ubl1 via a bipartite interaction involving two distinct linear motifs. The two proteins form a high-affinity complex implicating a hydrophobic helix, which docks into a hydrophobic groove on Ubl1, and a second interaction site in the proximity of N4. The strand between the two binding sites and the remainder of N3 remain flexible in the complex, and domains N1, N2, N4, and N5, as well as the SR region, are not directly implicated in the interaction. Nevertheless, the wrapping of N3 around Ubl1 leads to the massive compaction of the otherwise highly flexible N protein, suggesting that nsp3a is involved in trafficking and chaperoning N before encapsidation. Binding of short RNAs is also found to be abrogated in the compact Ubl1-bound form of N. The identification of linear motifs and binding sites that are important for viral function will provide active targets for the development of peptide-based viral inhibitors.

## RESULTS

### ITC identifies the thermodynamic nature of the interaction

Interaction between N and nsp3a was initially investigated using ITC. Different constructs of N were tested, comprising domains N123, N3, N234, N45, and short N3 (sN3, 191 to 263), in interaction with full-length nsp3a (1 to 206), Ubl1 (1 to 111), and short Ubl1 (sUbl1, 16 to 111) (Fig. 1, A and B). In all cases where interaction is observed, the thermodynamic nature of complex formation is conserved (fig. S1). The interaction exhibits dissociation constants in the range of 30 to 200 nM, depending on the exact constructs used (table S1). The enthalpic component dominates (20 to 30 kcal mol^−1^), with a much smaller and always negative entropic contribution (Fig. 1C and fig. S1). Very similar entropic and enthalpic contributions are measured for the complexes comprising constructs containing sN3 and Ubl1, suggesting that the interaction site present in sN3 dominates the thermodynamics of complex formation.

### NMR identifies the interaction interfaces of the dynamic complex

The backbone resonances of free N1 and N3 have been assigned (*32*), confirming the disordered nature of the two domains. NMR revealed the existence of a helical region in N3 (α_1_), spanning residues 219 to 230 with local propensities varying between 35 and 70% [1 to 2 ppm (parts per million) secondary C^α^ shift] and no secondary structure elsewhere (six residues in α_1_ could not be assigned in the free form of N3). With the exception of the first 15 residues, including the SR region, resonances from N3 superpose with those from N123, suggesting that N3 is equally flexible in the isolated form and in the presence of N1 and N2.

The backbone resonance assignment of nsp3a was also recently published (*53*), confirming the presence of a folded domain exhibiting the same secondary structure as the crystal structure of Ubl1 and a C-terminal domain that is essentially devoid of secondary structure. ^15^N relaxation confirms the disordered nature of this domain (fig. S2). The 15–amino acid N-terminal region of Ubl1 is also highly flexible in solution.

All NMR experiments measured on N:nsp3a complexes were hindered by substantial exchange broadening. Highest-quality spectra were recorded on complexes comprising an N-terminal truncated Ubl1 (sUbl1: 16 to 111) and sN3, molecular constructs that comprise all binding sites in both proteins (vide infra). Unless stated, these components were therefore used in subsequent assignment, relaxation, and structure determination experiments.

**Fig. 2.**
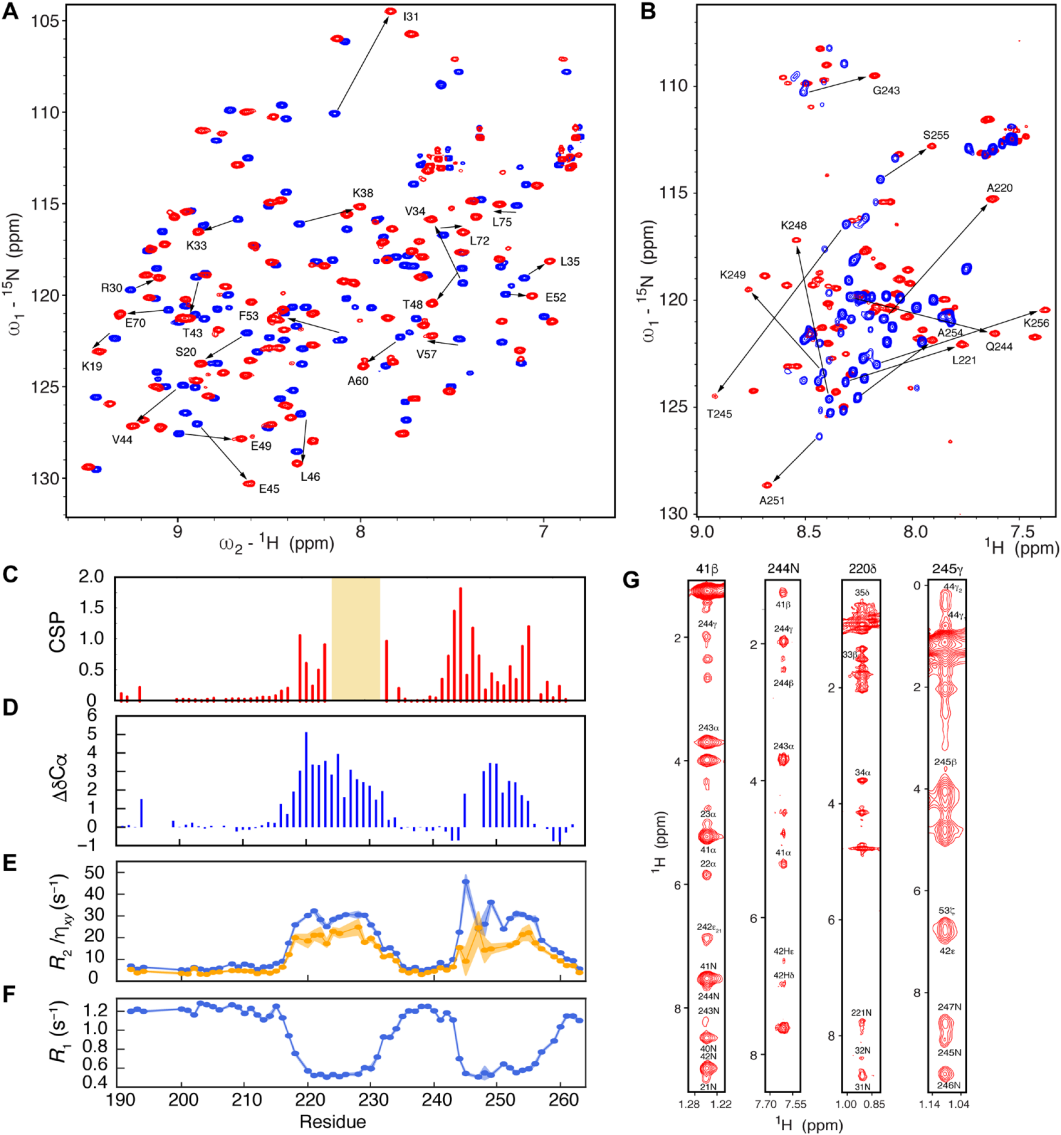
NMR characterization of the sN3:sUbl1 complex. (**A**) ^15^N-^1^H correlation spectra showing chemical shift perturbations (CSPs) of the sUbl1 spectrum upon binding of sN3. Exchange between free (blue) and bound (red) sUbl1 resonances is in the slow exchange regime. The spectrum shows a 1:1 complex at a concentration of 500 μM at 298 K and was measured at 850 MHz. (**B**) ^15^N-^1^H correlation spectra showing CSP of the sN3 spectrum upon binding of sUbl1. Exchange between free (blue) and bound (red) sN3 resonances is again in the slow exchange regime. The spectrum shows a 1:1 complex at a concentration of 500 μM at 298 K and was measured at 850 MHz. (**C**) ^15^N-^1^H CSP plotted as a function of sequence of sN3. Experimental conditions as in (B). Orange shading denotes the region of sN3 that could not be assigned in the free form of the protein. (**D**) Secondary structure propensity of sN3 in 1:1 complex with sUbl1. ^13^C^α^ chemical shifts of approximately +3.0 ppm correspond to a fully formed helix. (**E** and **F**) ^15^N spin relaxation of sN3 in 1:1 complex with sUbl1 (500 μM), measured at 950 MHz. Both transverse (*R*_2_, η*_xy_*) and longitudinal (*R*_1_) relaxation identifies two rigid binding sites separated by a highly flexible linker. (**G**) Sample strips from ^15^N- and ^13^C-edited NOESY-HSQC experiments, showing both inter- and intramolecular cross peaks, reporting on the structure of the sUbl1-sN3 complex. All mixing times shown were between 100 and 120 ms.

NMR chemical shift perturbations (CSPs) were measured to map the interaction sites between the two proteins (Fig. 2). The interaction is in the slow exchange regime on the NMR chemical shift time scale, compatible with the relatively tight affinity observed by ITC. CSPs in N3 are localized to two continuous regions of the IDR, helix α_1_ and the region following the poly-glutamine (polyQ) strand, spanning residues 243 to 255 (βα_2_) (Fig. 2C). Assignment of the backbone resonances of sN3 in complex with sUbl1 reveals that α_1_ binds in an α-helical conformation, while βα_2_ adopts a less well-defined conformation followed by a binding-induced α helix (Fig. 2D). ^15^N relaxation measured on sN3 in the presence of sUbl1 (Fig. 2, E and F) supports the existence of bipartite binding motifs, with elevated R_2_ and reduced R_1_ uniquely in α_1_ and βα_2_. The disordered regions, including the SR-rich and polyQ regions, therefore remain flexible in the complex and do not appear to be directly involved in binding.

Addition of sN3 to ^15^N-labeled sUbl1 reveals extensive CSPs that implicate helices α_A_ (33 to 39) and α_C_ (69 to 75), as well as the solvent-exposed strand of the β sheet and helix α_B_ (51 to 66) (fig. S3).

### Intrinsically disordered N3 folds around Ubl1 via two separate linear motifs

**Fig. 3.**
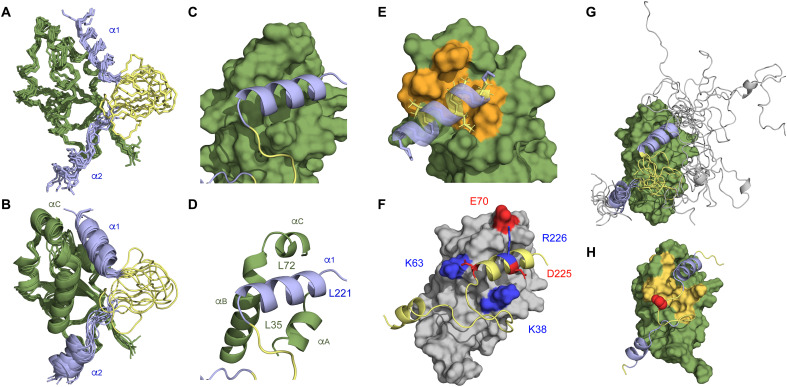
sN3 folds around sUbl1 upon binding via a bipartite binding motif. (**A** and **B**) Representation of the bundle of 10 structures determined from 594 experimental nOes measured on 1:1 complexes of sN3:sUbl1. Two binding sN3 sites are identified and shown in blue (sUbl1 in green). The first site (α_1_) binds in a hydrophobic groove. The second (βα_2_) binds to the solvent-accessible strand of the Ubl1 β sheet (^41^AYTVE^45^) and then folds into a helix located in the vicinity of the N terminus of Ubl1 helix α_B_. The disordered strand between the two binding sites on sN3 is shown in yellow. (**C** and **D**) Definition of the position of α_1_ (blue) in the hydrophobic groove situated between helices α_A_, the C terminus of α_B_ and α_C_ on sUbl1 (green). (**E**) Stabilization of the leucine-rich α_1_ helix of sN3 on sUbl1 via hydrophobic interactions with hydrophobic side chains on the surface of sUbl1 (orange shading). (**F**) Electrostatic stabilization of the interaction site. R225 and D226 on opposing sides of sN3 helix α_1_ point in the direction of E70 and K38, respectively, on either side of the groove. The terminal residue E231 is adjacent to K63. (**G**) Representation of the N3:Ubl1 complex that is in agreement with experimental SAXS data. The unfolded domain of N3 appears to transiently interact with the surface of Ubl1 to make a compact complex. (**H**) Representation of regions showing broadening on sUbl1 in the presence of spin-labeled C235. Orange surface represents region with PREs lower than 0.5. Position of C235 in the flexible loop is shown in red.

A series of ^15^N- and ^13^C-edited 3D NOESY (nuclear Overhauser effect spectroscopy) experiments were performed to identify inter- and intramolecular contacts (Fig. 2G). Although exchange occurring on different time scales affected spectral quality, it was possible to extract both intra- and intermolecular distance restraints that allowed us to determine the structure of the complex. Approximately 50% of side chains were assigned, and 54 intermolecular distance restraints and 543 intramolecular restraints were extracted. The alpha variant of SARS-CoV-2 comprises a mutation at position S235F in N. CSPs between sN3 and sUbl1 are not affected by this mutation (fig. S4), although NOESYs were slightly higher quality. Data were therefore combined with those of the ancestral form to determine the structures of the complex. Additional distance information was derived from site-specific ^13^C labeling of A220I (Fig. 2G). The Ubl1 domain is slightly better defined [root mean square deviation (rmsd), 1.41 ± 0.26 Å] compared to the two binding sites (1.80 ± 0.42 Å) (Fig. 3, A and B). The structured part of the complex, defined as Ubl1(17 to 106), N(219 to 232), and N(245 to 254), has a backbone rmsd of 0.70 Å with respect to the medoid structure.

The complex is mediated by two linear motifs of N3, the first comprises the central helix α_1_, that binds in the hydrophobic groove between α_C_ (65 to 70) and α_A_ (33 to 39) of Ubl1 (Fig. 3, C and D). The second motif, 13 amino acids downstream of α_1_, follows the polyQ strand and comprises a QTVT (glutamine-threonine-valine-threonine) motif followed by six residues (249 to 255) that fold into an α-helical conformation (α2) upon binding, located in the vicinity of the loop between the β sheet and the N terminus of Ubl1 helix α_B_. These same residues are seen to form an α helix in the N-terminal region of the crystal structure of N4 (*24*, *28*) and were shown to adopt an α-helical conformation in solution studies of N4 (*55*). The structure of sUbl1 compares closely with the crystal structure (PDB 7kag) of Ubl1 (1.7 ± 0.2 Å; fig. S5), with the main differences located in the C and N termini of helices α_A_ and α_C_, respectively.

The α_1_ N3 interaction site is stabilized by extensive hydrophobic contacts with Ubl1. Methyl groups in the N-terminal poly-leucine motif (^219^LALLLL^224^) of α_1_ interact with hydrophobic residues at the base of the groove, partially burying the helix (Fig. 3E) such that mainly polar and charged residues are exposed to the solvent. R225 and D226 of N3, situated on opposing sides of helix α_1_, point in the direction of Ubl1 E70 and K38, respectively, on either side of the groove, while the terminal residue E231 is adjacent to K63, suggesting that electrostatic interactions also play an important role in stabilizing binding (Fig. 3F). The second binding site involves the polar strand ^244^QTVT^247^ of N3, which binds in a weakly defined extended conformation, adjacent to the external β strand in Ubl1 (41 to 44), similar to mechanisms adopted by small ubiquitin-like modifier (SUMO)–interacting motifs (*56*).

### Cooperative binding of the two linear motifs forming a highly dynamic complex

In total, the two interaction sites implicate a stretch of around 40 N3 amino acids when binding to Ubl1, although NMR spin relaxation shows that the two binding motifs are decoupled by a highly dynamic N3 linker (234 to 243). Exchange spectroscopy (EXSY) experiments also reveal the presence of slow exchange of the second binding motif for nine consecutive amino acids (243 to 251) between free and bound states, on a time scale of 40 to 100 s^−1^ (figs. S6 and S7 and Materials and Methods). Slow exchange is not seen for the first helix, indicating that the second binding site exchanges between free and bound states, while α_1_ is bound, consistent with the α_1_ site having a higher affinity, such that binding of the second site (βα_2_) is cooperative and dependent on binding of α_1_.

SAXS was also measured on the N3:Ubl1 complex. An ensemble of conformations was generated, on the basis of the NMR bundle with the remaining region of N3 (175 to 215) sampling coil conformations in agreement with NMR chemical shifts and relaxation. This pool of conformers represents a more extended complex than measured experimentally (fig. S8). Selection of a sub-ensemble of conformers in agreement with the experimental SAXS data demonstrates that the complex is more compact, apparently involving additional transient contacts between the region 175 to 215 and the surface of Ubl1 (Fig. 3G and fig. S8).

### Validation of the sN3:sUbl1 structure

The orientation of helix α_1_ in the hydrophobic groove was independently verified using paramagnetic tags attached to cysteine labels in the flexible 232 to 243 linker (M234C and S235C), resulting in broadening observed on Ubl1 in the concave face formed between helices α_A_ (33 to 39) and α_C_ (65 to 70) and the β sheet (Fig. 3H and fig. S9). In addition, a truncated construct of sN3 (215 to 263) showed indistinguishable affinity for sUbl1 but induced slight CSPs relative to the longer construct, located uniquely on the opposing face of the protein (fig. S10), again supporting the determined orientation of the helix.

### Interaction with Ubl1 results in large-scale compaction of N

**Fig. 4.**
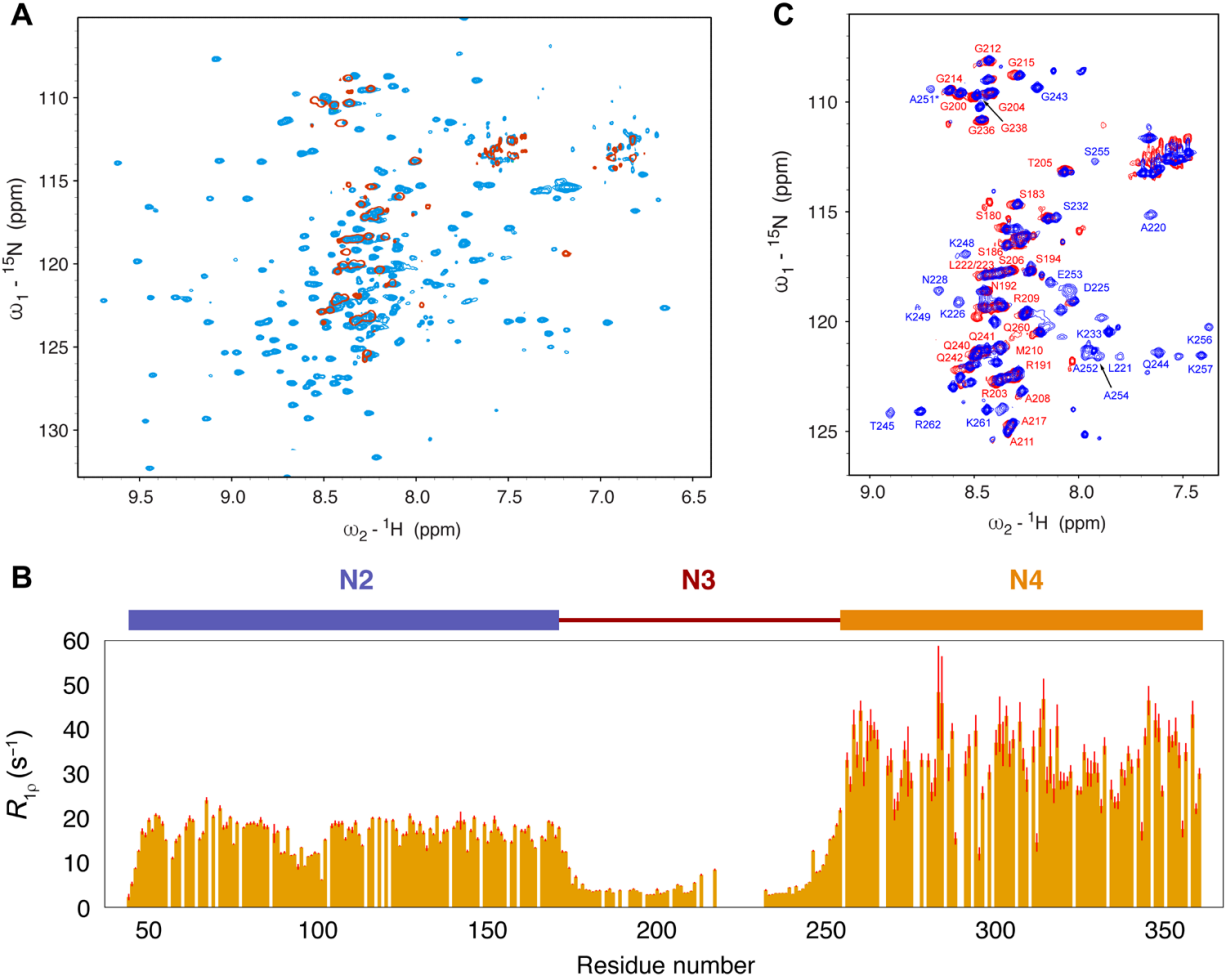
Dynamics of N234 in free and Ubl1-bound forms. (**A**) ^15^N-^1^H correlation spectrum showing free (light blue) and Ubl1-bound (red) dimeric N234. All signals from N2, N4, and the two binding sites (α_1_ and βα_2_) are broadened beyond detection when bound to Ubl1. Spectra recorded at 850 MHz and at 298 K. (**B**) Transverse ^15^N spin relaxation measured on free N234 (850 MHz, 380 μM), showing characteristic dynamic properties of the three domains. N4 exhibits the slowest rotational correlation time, while N2 retains substantial degrees of conformational flexibility relative to this, via the dynamic behavior of N3, which behaves like an intrinsically disordered domain. (**C**) Comparison of the sN3-sUbl1 and N234-sUbl1 1:1 complexes. The remaining observable resonances can be identified to correspond to the flexible regions of N3 (from 175 to 215 and 232 to 242).

We investigated the structural nature of longer constructs of N and the impact of the interaction with Ubl1 on intrinsic conformational sampling. NMR spectroscopy of dimeric N234, comprising both folded domains (636 amino acids in total), exhibits a well-resolved ^15^N-^1^H correlation spectrum (Fig. 4A), allowing measurement of spin relaxation that identifies distinct dynamic properties of N2, N3, and N4 in the context of the longer construct (Fig. 4B). Not surprisingly, the RNA binding domain, N2, exhibits increased dynamics (lower transverse relaxation rates) compared to the dimerization domain N4, which shows the highest relaxation rates, while the disordered linker N3 exhibits the highest level of dynamics (considerably lower relaxation rates). The chemical shifts of N2 and N4 are indistinguishable from the chemical shifts of the isolated domains (fig. S11) (*25*, *55*). These observations are consistent with the perception of N234 as folded domains tethered by a highly flexible linker (sampling statistical coil conformations except for the α_1_ helical element), with no significantly populated interaction between these domains in solution.

We investigated the interaction of Ubl1 with dimeric N234, forming a complex of 860 amino acids in total, by observing ^15^N-labeled N234 in complex with unlabeled Ubl1. Although ITC measurements show a slightly reduced affinity (Fig. 1), possibly due to reduced accessibility of N3 to the binding site in the presence of N2 and N4, the interaction is maintained in the context of this longer construct, with only amino acids preceding the α_1_ binding site and between the α_1_ and βα_2_ binding sites remaining visible by NMR (Fig. 4, A and C). The remainder of the complex is undetected under our conditions, consistent with binding to a much larger object, causing extreme line broadening of amide resonances from N2, N4, and the two binding sites.

**Fig. 5.**
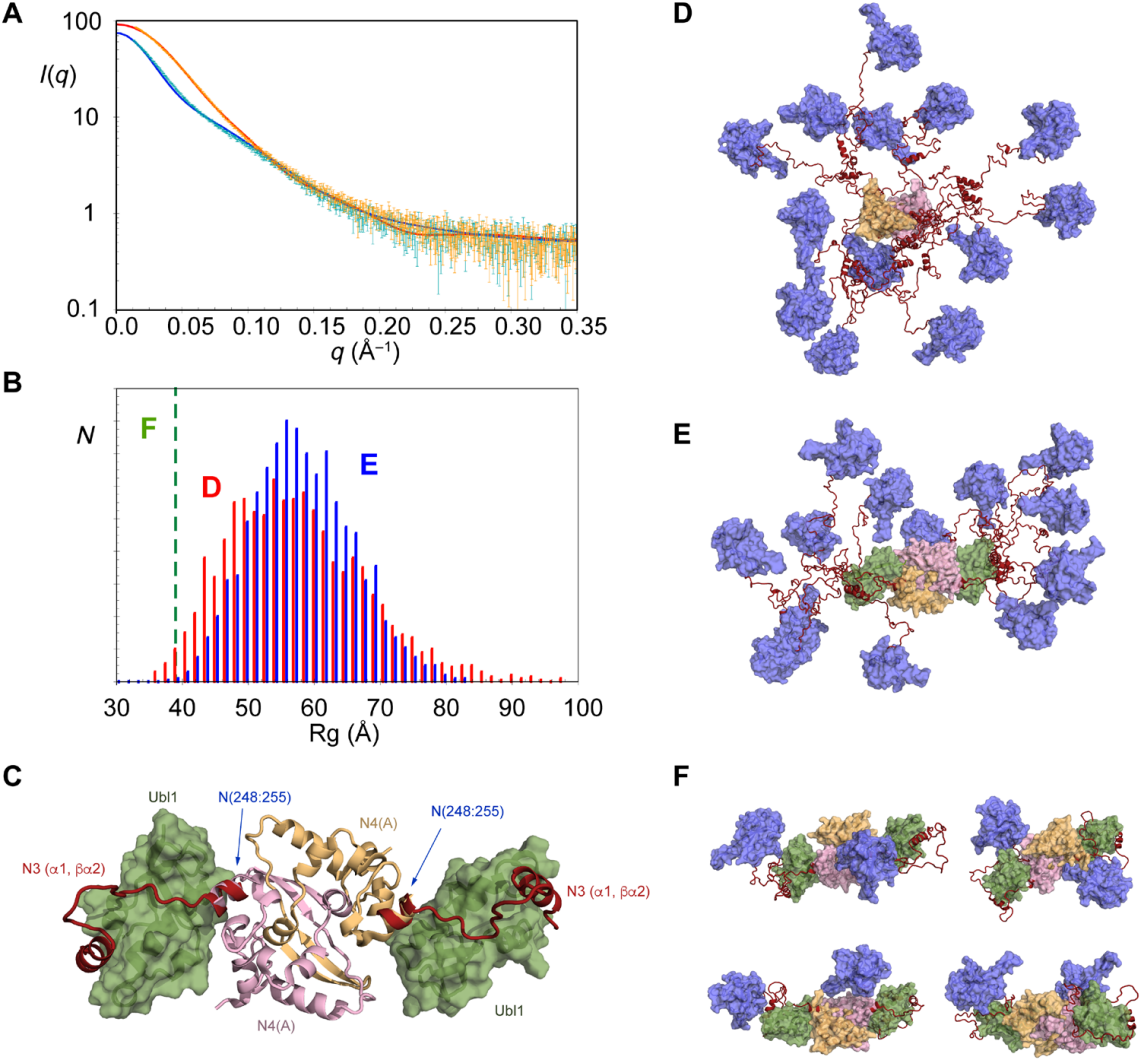
Binding of Ubl1 causes massive compaction of N234. (**A**) SAXS of N234, free (green) and Ubl1-bound (orange). Experimental data are broadly reproduced by a model with N3 sampling statistical coil conformations [see (E)] [blue curve (D)]. Binding of Ubl1 provokes a collapse in conformational sampling. These data can only be reproduced by compact conformations of N234:Ubl1 [examples in (F)] shown by the red curve. (**B**) Distribution of radii of gyration (Rg) of 10,000 models of N234 (red), randomly sampling conformational space defined by statistical coil sampling for N3 (except helix α_1_) (D). Blue: Distribution Rg of 10,000 models of N234 bound to Ubl1, randomly sampling conformational space defined by statistical coil sampling for the dynamic region of N3 [175 to 215 and 232 to 242 (E)]. Green dashed line shows the Rg measured from experimental SAXS, in agreement with highly compact models of N234:Ubl1 complex (F). (**C**) Representation of the core scaffold, comprising the sN3:sUbl1 complex and N4 dimer [PDB code 6wzo (*28*)]. Relative positions of N4 and sN3:sUbl1 were assembled by superposition of helix α_2_. (**D**) Statistically available conformational sampling of free N234 described in (B). Colors as in Fig. 1: magenta, N2 domains; pink, N4; orange, N4; red, N3. (**E**) Statistically available sampling of Ubl1-bound N234 described in (B). Colors as in (D): green, Ubl1. (**F**) Models that reproduce experimental SAXS data of Ubl1-bound N234. On the basis of our current experimental data, we cannot distinguish between different compact conformations. Color legend as in (E).

The dynamic model of the unbound N234 dimer is supported by SAXS (Fig. 5A). The conformational behavior of free N234 was modeled using crystal structures of N4 (*57*) and N2 (*24*) and sampling of N3 in agreement with NMR chemical shifts (Fig. 5D). SAXS data were predicted from the ensemble of conformers representing this state. This direct comparison (nonfitted) broadly reproduces experimental data (Fig. 5A) (the data suggest that the ensemble may even be slightly more extended than this), again demonstrating that all experimental data measured on the unbound N234 dimer are consistent with extensive conformational sampling of the linker domain with negligible contacts between N2 and N4 (*29*). The distribution of simulated radii of gyration varies in a range from 40 to 95 Å with a maximum population around 52 Å (Fig. 5B).

On the basis of SAXS, binding of dimeric N234 to Ubl1 provokes a major reduction of radius of gyration (Fig. 5A) (from 50 to 39 Å according to Guinier analysis). The theoretically accessible conformational sampling of Ubl1-bound N234 was modeled by superimposing the common α_2_ helix from our NMR ensemble and the crystal structure of N4 (Fig. 5C), the crystal structure of N2, and building the flexible, NMR-visible domains (174 to 217 and 231 to 243) (Fig. 5, D and E). Ten thousand such conformers were initially compared individually to the experimental SAXS data. SAXS from the bound state can only be reproduced by highly compact individual models of the complex, exhibiting interdomain contacts between N2 and either Ubl1 or N4 (Fig. 5F). SAXS is not capable of distinguishing between such models, and the level of domain flexibility within this framework is still substantial in particular, N(174 to 217) and (231 to 243) remain highly dynamic. Notably, however, the available degrees of conformational freedom of the flexible region of N3 in the N234:sUbl1 complex would in theory allow a broad distribution of radii of gyration (Fig. 5B), which is evidently not populated in solution. On the basis of the calculated distribution, the probability of such compact conformations being sampled by unbiased statistical coil sampling of the flexible region of N3 is estimated to be less than 1 in 10^3^. While further detail of the physical basis of this compaction will require additional interdomain information, these data unambiguously reveal that Ubl1 binding results in a massive collapse of the conformational space available to N234.

We also observe CSPs and local NMR relaxation induced in the disordered domain of nsp3a in the presence of N234 (fig. S12). This indicates that the charged regions in the acidic domain of nsp3a interact transiently with N234, possibly implicating the SR region of N3 or RNA binding sites on N2 and N4 (*57*).

### Formation of the Ubl1:N complex abrogates binding to short RNA strands

**Fig. 6.**
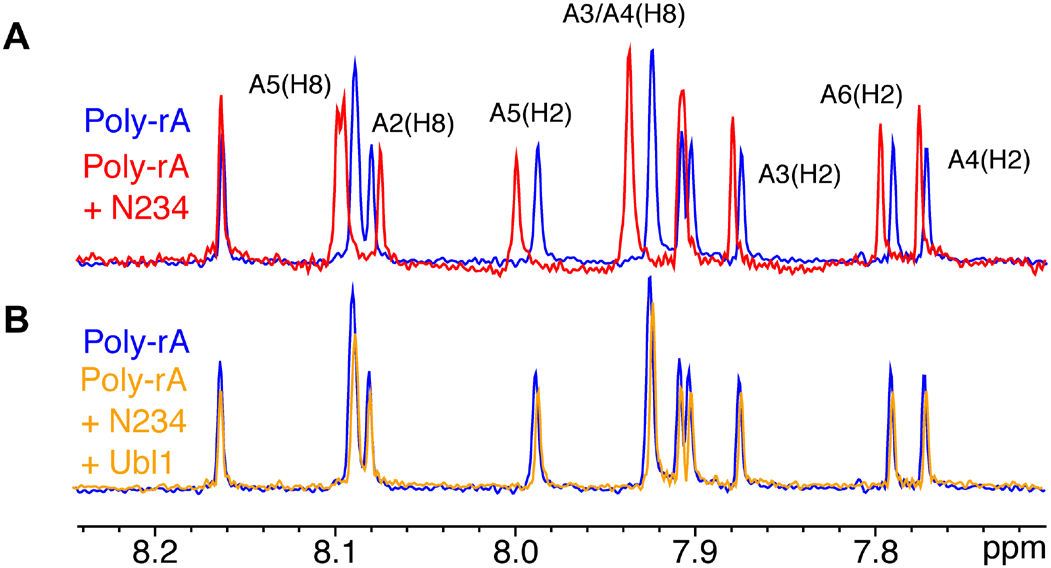
Formation of the N234-Ubl1 complex abrogates binding of short RNAs. (**A**) 1D NMR spectra of 100 μM polyA hexameric RNA free (blue) and upon addition of 50 μM N234 (red). Clear chemical shifts report on binding of RNA to N234. (**B**) One-dimensional NMR spectra of 100 μM polyA hexameric RNA free (blue) and upon addition of 50 μM N234-Ubl1 complex (orange). Formation of the complex abrogates binding.

One of the key roles of N in viral function is to bind the viral RNA genome, forming nucleocapsid-like structures. We have investigated the impact of complex formation with Ubl1 on RNA binding to the N234 construct by adding short, polymeric RNA (polyadenosine, polyA). Interaction of RNA with N234 is observed by 1D NMR (Fig. 6A), an interaction that is abolished in the presence of Ubl1 (Fig. 6B).

## DISCUSSION

The molecular mechanisms underlying the function, intracellular transport, and molecular colocalization of the different components of the replication machinery of SARS-CoV-2 remain poorly described but represent major targets for inhibitory strategies to treat COVID-19. Using NMR, SAXS, and ITC, we describe the dynamic interaction between nsp3a and N from SARS-CoV-2 that plays a vital role in this process. Nsp3 is one the viral proteins that are critical for formation of DMVs (*54*), while N is essential for genome encapsidation, protection of the viral genome from host immunity, as well as regulation of replication (*8*) and transcription (*9*). Nsp3 is thought to play a role in trafficking N to the RTC and colocalizes with N in the vicinity of DMVs (*12*). In MHV, N and nsp3a were shown to interact (*15*, *16*, *50*), an interaction mediated by the Ubl1 domain (*15*), which was also shown to be essential for viral replication.

ITC demonstrates that N1, N2, N4, and N5 do not contribute significantly to the binding enthalpy or entropy of interaction between N and nsp3a, identifying N3 and Ubl1 as the essential components. The interaction has a relatively strong affinity, exhibiting dissociation constants between 30 and 200 nM depending on the exact constructs, and is dominated by enthalpic contributions. The essential N3 domain comprises a long intrinsically disordered SR-rich N terminus and a polar C terminus, flanking a central hydrophobic strand that exhibits strongly helical propensity (α_1_), which appears to be conserved over betacoronaviridae (*58*).

NMR CSP and spin relaxation map the interaction site of N for Ubl1 to two distinct linear motifs in the central and C-terminal regions of N3. The conformation of Ubl1 resembles that of the recently determined crystal structure (PDB code 7kag) and the NMR structures of SARS-CoV (*49*) and MHV (*50*), with local differences in the N-binding sites. The structure of the complex reveals a bipartite interaction, which folds N3 around Ubl1, with the α_1_ helix binding in the hydrophobic groove formed between helices α_C_ (65 to 70) and α_A_ (33 to 39). The α_1_ interaction is further stabilized by electrostatic contacts with surface residues of Ubl1. The extent of intermolecular interaction corroborates the strongly enthalpic nature of complex formation. The α_1_ binding site is linked to the second (βα_2_) binding site via a flexible linker. βα_2_ forms an extended conformation that binds to the external strand of the β sheet of Ubl1 (41 to 44) and comprises a final helix whose orientation is less well defined. α_2_ coincides with the short helix found in the N terminus of the crystal structure of N4, suggesting that Ubl1 and N4 would lie in close proximity in the complex of dimeric N234 with Ubl1. In summary, α_1_ and βα_2_ mutually engage to form a bipartite interaction, folding N3 around Ubl1. The expected sampling radius of the remaining N-terminal SR-rich domain is also reduced, apparently due to transient interactions with Ubl1.

The βα_2_ site appears to bind weaker than α_1_, as revealed by the presence of exchange between bound and free states, while α_1_ remains bound, suggesting cooperative binding driven by the α_1_ binding site. We note that slow conformational exchange was also recently observed in helix α_2_ in the context of N4 in solution (*55*). It is not clear what role the two distinct binding sites play, although freeing the βα_2_ site of nsp3a temporarily from the surface of N4 would allow extensive remodeling of the complex, for example, upon RNA binding.

**Fig. 7.**
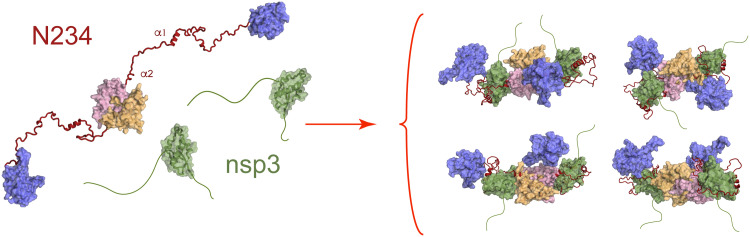
Impact of binding of nsp3a on the conformational sampling of N234. Cartoon representation of the collapse in conformational sampling that occurs upon folding and binding of N3 on the surface of Ubl1, accompanied by further collapse of the position of N2 in the vicinity of the core scaffold formed by N3, N4, and Ubl1. Color legend as in Fig. 1: magenta, N2 domains; pink, N4; orange, N4; red, N3; green, nsp3a.

NMR measurements of domains N234, which is dimeric in solution, reveal a flexible multidomain molecule, with the RNA binding (N2) and dimerization (N4) domains connected by highly dynamic N3 linker domains. SAXS of the N234:Ubl1 complex reveals that Ubl1 binding causes a massive contraction of the conformational space sampled by dimeric N234 in solution (Fig. 7). Modeling of the possible conformational sampling of N234 in the N234:Ubl1 complex demonstrates that the N2 domains must lie predominantly in close proximity to the folded domains of N4 or Ubl1. Such conformations are statistically highly unlikely when the remainder of N3 is flexible in the complex and therefore must be stabilized by as-yet unidentified interactions between N234 and Ubl1 domains. NMR data demonstrate that the complex, while compact, still exhibits considerable flexibility, with the linker regions, including the SR region and the polyQ strand, remaining highly dynamic. It is not clear whether the exchange of the βα_2_ binding site is maintained in the large complex, although this would be compatible with the existence of visible signals in the loop between α_1_ and βα_2_.

It is interesting to speculate on the role of this interaction in trafficking N to the RTC. Two colocalization processes are thought to accompany replication of the viral genome and transcription of subgenomic regions. First, replication of betacoronaviral RNA has been shown to occur principally in DMVs (*5*, *6*), formed from remodeling of host membranes upon interaction with viral proteins, including nsp3. A recent study using electron tomography revealed the presence of molecular pores in DMVs and identified nsp3 as a constituent of the architecture of the pore (*4*). Nsp3a was located at the terminus of prongs extending into the cytosolic side of the pore, with six copies of the protein protruding away from the pore at symmetry-related positions on the rim of the channel. Increased density in electron tomograms suggested accumulation of N localized at the exterior of the DMV, possibly to allow rapid encapsidation and/or participation in the RTC. In view of the narrow dimensions of the exit channel, it seems plausible that naked genomic RNA may exit the DMVs following synthesis, with encapsidation by N occurring in the immediate vicinity of the exit channel. An interaction between N and nsp3a may facilitate this process.

Second, N has been shown to colocalize in the vicinity of the RTC, forming membraneless organelles, either in the presence of viral RNA or under certain conditions in its isolated form. This process of LLPS has been associated with colocalization of other components of the replication machinery, for example, the RNA polymerase was recently found to partition into membraneless organelles comprising N and RNA (*34*). Although it is not yet known how droplet formation may be related to the formation of DMVs and SARS-CoV-2 replication, the concentration of components of the RTC may present advantages in terms of RNA synthesis and protection from host defense strategies, as well as efficient encapsidation. In this respect, it is interesting to note that Ubl1 can be recruited into droplets formed by N in vitro (*39*), suggesting a mechanism by which a highly concentrated reservoir of N could be maintained in the vicinity of the DMV pore. It has recently been suggested that the region comprising residues 210 to 246 is essential for phase separation (*40*) and that N4 and residues 210 to 246 provide the multivalency associated with LLPS. This is intriguing because this latter region coincides partially with the two linear motifs forming the interaction surface identified here (218 to 255), and tight interaction with nsp3a might be expected to interfere with the network of weak interactions stabilizing the droplet.

The remarkable transformation of the highly flexible N, into a much more compact conformation upon binding Ubl1, may suggest a role for nsp3 as a chaperone of N before encapsidation of the viral genome at the replication site, similarly to the role of phosphoproteins in the negative-sense RNA paramyxoviridae (*59*). This chaperoning role could be related to the observed abrogation of RNA binding upon formation of the complex. Our experimental data demonstrate that the behavior of the N2 domains is strongly affected by formation of the compact complex with nsp3a, placing them in the vicinity of the N34:Ubl1 core of the complex and possibly protecting available RNA binding sites. It is also possible that nsp3a binding inhibits assembly of N into higher-order structures that form the nucleocapsids. In this dynamic complex, the long disordered acidic tail of nsp3a also interacts transiently with either N4 or N2, a mechanism that may provide a protection against nonspecific, or untimely, binding to RNA.

In conclusion, we have determined the structure of the molecular complex between SARS-CoV-2 N and nsp3 proteins, which are both associated with viral replication and transcription. We identify two distinct linear motifs that combine to wrap N3 around Ubl1, resulting in substantial collapse in the dimensions of N when bound to nsp3a. N3 remains locally dynamic, with the β_1_α_2_ site exchanging between free and bound conformations while α_1_ is bound. Combination with the recently published structure of N4 dimer allows us to propose a model for the compaction of N resulting from interaction with Ubl1, providing a mechanism of chaperoning or colocalization of N before encapsidation. Formation of this complex regulates binding to RNA, which may again play a chaperoning role in trafficking of N to the RTC. These observations, allied with the ability of nsp3a to localize in condensates formed by N, allow us to speculate on the role of the interaction in the replication cycle. The identification of two linear motifs and two interaction surfaces that are important for viral function provides previously unidentified active targets for the development of peptide-based strategies to combat COVID-19.

## MATERIALS AND METHODS

### Protein expression and purification

All proteins were expressed in *Escherichia coli* BL21 (DE3) Novagen) or AI (Invitrogen). All N constructs, N3 (175 to 263), sN3 (191 to 263), xsN3 (215 to 419), N45 (255 to 419), N (1 to 419), and N234 (47 to 364), were cloned into a pESPRIT vector between the AatII and Nott1 cleavage sites with His_6_-tag and TEV (tobacco etch virus) protease cleavage sites at the N terminus (GenScript Biotech, The Netherlands). Single-point mutations (sN3 A220I, M234C, S235C, and S235F) were made in-house by site-directed mutagenesis. Transformation was performed by heat shock, and proteins were expressed in *E. coli* AI cells (Thermo Fisher Scientific) for 5 hours at 37°C after induction at an optical density of 0.6 with 1 mM isopropyl-β-d-thiogalactopyranoside (IPTG). Cells were harvested by centrifuging at 5000 rpm and resuspended in 20 mM tris (pH 8.0) and 500 mM NaCl buffer; 1 M NaCl was used for N1–419, lysed by sonication, and centrifuged again at 18,000 rpm at 4°C. The supernatant was subjected to standard Ni purification. Proteins were eluted with 20 mM tris (pH 8), 500 mM NaCl, and 500 mM imidazole. Samples were then dialyzed against 20 mM tris (pH 8), 500 mM NaCl, and 5 mM 2-mercaptoethanol at room temperature overnight. Following TEV cleavage and removal of the excess N-terminal tag and TEV by Ni affinity, samples were concentrated and subjected to size exclusion chromatography (SEC; Superdex 75/200) in 50 mM Na-phosphate (pH 6.5), 250 mM NaCl, and 2 mM dithiothreitol (DTT) buffer (NMR and ITC buffer).

The primary sequence of nsp3a from SARS-CoV-2 was extracted from National Center for Biotechnology Information (NCBI) genome entry NC_045512.2 [GenBank entry MN908947.3], and a gene codon-optimized for expression in *E. coli* was commercially synthesized (GenScript) and subcloned in a pET21b(+) vector. Two constructs corresponding to residues 1 to 111 (Ubl1) or 1 to 206 (nsp3a) were synthesized. Hexahistidine, GST, and TEV cleavage tags were introduced at the N terminus for the purposes of protein purification. sUbl1 (16 to 111) was cloned into a pESPRIT plasmid as described for the N constructs. The plasmids were transformed into BL21 (DE3) *E. coli* cells. Cells were grown at 37°C until OD_600_ (optical density at 600 nm) of 0.6 to 0.8, induced with IPTG, and incubated for 12 hours at 22°C. Bacteria were harvested by centrifugation, and the cell pellet was resuspended in buffer A [50 mM tris-HCl (pH 8.0) and 150 mM NaCl] with protease inhibitors (cOmplete, Roche). Cell lysis was performed by sonication, followed by centrifugation (45 min, 18,000 rpm at 5°C). Supernatant was subjected to a standard Ni purification. The protein was purified by affinity chromatography on Ni-NTA agarose (Thermo Fisher Scientific), washed with buffer A supplemented with 20 mM imidazole, and eluted with buffer A supplemented with 500 mM imidazole. Following TEV cleavage, samples were concentrated and subjected to SEC with a Hiload 16/600 Superdex 75 column (GE Healthcare) in NMR buffer [50 mM Na-phosphate (pH 6.5), 250 mM NaCl, 2 mM DTT]. For ^15^N and ^13^C isotope labeling, cells were grown in M9 minimal medium supplemented with ^15^N-NH_4_-Cl and ^13^C_6_-d-glucose. For selective labeling of the isoleucine Cδ1 methyl group in the sN3 A220I mutant, 2-ketobutyrate-4-^13^C (50 mg/liter; Sigma-Aldrich) was added to unlabeled M9 medium 1 hour before induction (*60*).

### Isothermal titration calorimetry

ITC was measured using MicroCal iTC200 (GE healthcare) at 25°C. The titration experiments were performed by adding 2.0 μl of aliquots of 200 μM of the different N constructs (sN3, N3, N45, N234, and N123) into the microcalorimeter cell filled with 20 μM of sUbl1 (16 to 111), Ubl1 (1 to 111), or Nsp3a (1 to 206). N constructs were titrated through 20 injections at 180-s intervals; the reaction mixture was continuously stirred at 750 rpm. The titration curve was fitted to the experimental data using Origin version 7.0 with the ITC plugin from MicroCal. All experiments were performed with both components in NMR and ITC buffer (see above).

### NMR experiments

All NMR experiments were done in 50 mM Na-phosphate (pH 6.5), 250 mM NaCl, and 2 mM DTT or 50 mM Na-phosphate (pH 6.5), 250 mM NaCl, and 1 mM tris(2-carboxyethyl)phosphine (TCEP) at 25°C unless stated otherwise. Experiments were acquired on Bruker spectrometers with ^1^H frequencies of 600, 700, 850, 950, and 1000 MHz. Spectra were processed with NMRPipe (*61*) and visualized using NMRFAM-Sparky (*62*).

^15^N R_1ρ_ relaxation was measured at 298 K and a ^1^H frequency of 950 MHz using a spin lock of 1.5 kHz (*63*). Typical relaxation delays of 1, 20, 50, 70, 70, 90, 120, 160, and 200 ms and 0, 0.08, 0.2, 0.4, 0.6, 0.6, 0.8, 1.04, 1.4, and 1.8 s were used for ^15^N R_1ρ_ and ^15^N R_1_ and included repetition of one delay. Relaxation rates were fitted using in-house software, and errors were estimated with noise-based Monte Carlo simulation.

Paramagnetic relaxation enhancement (PRE) effects were measured from the peak intensity ratios by comparing ^15^N-heteronuclear single-quantum coherence (HSQC) 2D spectra recorded on samples labeled with 2,2,6,6-tetramethylpiperidine 1-oxyl (TEMPO) and samples reduced by the addition of 2 mM ascorbic acid. Cysteine mutants of sN3 at positions 234 and 235 were reduced with 10 mM DTT at 4°C for 12 hours and dialyzed into 50 mM phosphate buffer (pH 7.0) containing 250 mM NaCl without DTT. Tenfold molar excess of 4-maleimido-TEMPO dissolved in dimethyl sulfoxide was added to the reduced cysteine mutants. The reaction was incubated for 2 hours at room temperature and then dialyzed into NMR buffer at 4°C for 12 hours to eliminate the excess of TEMPO.

^13^C- and ^15^N-edited 3D NOESY-HSQC experiments (*64*) were run with ^15^N and ^15^N, ^13^C-labeled sN3 in 1:1 complex with unlabeled sUbl, with ^15^N and ^15^N, ^13^C-labeled sUbl1 in 1:1 complex with unlabeled sN3 and ^15^N, ^13^C, ^2^D-labeled sN3 in 1:1 complex with unlabeled sUbl, and ^15^N and ^15^N, ^13^C, ^2^D-labeled Ubl1 in 1:1 complex with unlabeled N3. ^13^C-edited NOESY-HSQC was performed on selectively ^13^C isoleucine δ1 methyl-labeled A220I mutant of sN3, and 2D NOESY experiments were performed on sUbl1 in 1:1 complex with sN3. NOESY mixing times of 75, 100, 120, and 150 ms were used. Side-chain assignment was achieved using HCC(CO)NNH (*65*) and HCCH-TOCSY (*66*) experiments.

Backbone resonance assignment of resonances of the complex was initially obtained on ^15^N, ^13^C, ^2^H-labeled samples of N3 and Ubl1 in 1:1 complex with their respective unlabeled partners, using band-selective excitation short-transient (BEST)–type triple resonance experiments (*67*). These assignments were directly transferrable to the shorter sN3 and sUbl1 constructs. To obtain a complete assignment of the α_1_ helix of sN3 in the complex, the S235F mutant of sN3 was used, for which 3D correlation spectra were of highest quality. ^13^C^α^ chemical shifts were compared to random coil values using the program SSP (*68*).

Statistical analyses of the NMR ensemble were carried out by wwPDB dedicated software upon structure deposition in the PDB and Biological Magnetic Resonance Data Bank (BMRB). NMR structures have been deposited in the Brookhaven PDB under the accession code 7pku. Assignment of NMR resonances has been deposited in the BMRB under the accession codes 51052 and 34661.

### NMR EXSY

An NOE buildup curve was measured by repeating a 2D NOESY experiment with the mixing time varying from 10 to 700 ms. The NOE buildup was followed by measuring the intensity of resolved exchange cross peaks and fitted using standard expressions describing longitudinal exchange processes (*69*). Buildups of nine exchange cross peaks associated with residues in the βα_2_ binding site were fitted simultaneously, extracting common apparent exchange rate of 42 ± 13 s^−1^ and ^1^H longitudinal relaxation rate (R_1_) of 18 ± 3 s^−1^. Using expressions in (*69*) to account for the effect on the low (~1%) population of unbound βα_2_ on the apparent exchange rate, we estimate that on-off dynamics of the βα_2_ binding site is characterized by a rate constant *k*_ex_ ~ 100 s^−1^.

### Small-angle x-ray scattering

Before measurement, concentrated samples were dialyzed into fresh NMR buffer overnight. Samples of 1:1 complexes of N3(175 to 263):Ubl1(1 to 111) and N234(47 to 364):Ubl1(1 to 111) and N234(47 to 364) were diluted to 0.5, 1.0, and 2.0 mg/ml. SAXS measurements were acquired at the European Synchrotron Research Facility in Grenoble, France, on the beamline BM29 (see table S3) (*70*).

### Structure determination

NMR structure calculations were performed using CNS software, using previously described simulated annealing and restrainted molecular dynamics protocols (*71*). Restraints were identified from ^15^N and ^13^C NOESY HSQC spectra in the presence of unlabeled partner proteins. Backbone dihedral angles in secondary structural elements were identified from ^13^C secondary chemical shift analysis, and hydrogen bonding in β sheet of sUbl1 was identified from interstrand nOes. Statistical analyses of the NMR ensemble were carried out by wwPDB dedicated software upon structure deposition in the PDB and BMRB.

SAXS data measured on N3:Ubl1 were simulated by constructing disordered regions (residues 1 to 15 for Ubl1 and 175 to 217 and 258 to 263 for N3) onto the 10 members of the NMR ensemble (from residues 218 to 257 for N3), using the statistical coil algorithm flexible-meccano (*72*). SAXS curves were predicted using the program Crysol (see table S3) (*73*). Ensembles of conformations were selected from a pool of 10,000 such structures of the Ubl1:N3 complex using the algorithm ASTEROIDS (*74*). Data were also calculated from the entire ensemble for comparison. SAXS data measured on N234:Ubl1 were simulated by first constructing the core scaffold of the complex, comprising the sN3:sUbl1 complex and N4 dimer (PDB code 6wzo) (*28*). The relative positions of N4 and sN3:sUbl1 were assembled by superposition of helix α_2_. The procedure was repeated for each of the 10 NMR models that have slightly different helical orientations. Disordered regions of N3 (175 to 217) were built using the statistical coil algorithm flexible-meccano (*72*) and terminated with the known structure of N2. Twenty thousand conformations were individually compared to experimental SAXS data using Crysol (table S3) (*73*).

## Notes

View/request a protocol for this paper from *Bio-protocol*.

## References

[1] Y. Gao, L. Yan, Y. Huang, F. Liu, Y. Zhao, L. Cao, T. Wang, Q. Sun, Z. Ming, L. Zhang, J. Ge, L. Zheng, Y. Zhang, H. Wang, Y. Zhu, C. Zhu, T. Hu, T. Hua, B. Zhang, X. Yang, J. Li, H. Yang, Z. Liu, W. Xu, L. W. Guddat, Q. Wang, Z. Lou, Z. Rao, Structure of the RNA-dependent RNA polymerase from COVID-19 virus. Science 368, 779–782 (2020). 32277040 10.1126/science.abb7498 PMC7164392

[2] H. S. Hillen, G. Kokic, L. Farnung, C. Dienemann, D. Tegunov, P. Cramer, Structure of replicating SARS-CoV-2 polymerase. Nature 584, 154–156 (2020). 32438371 10.1038/s41586-020-2368-8

[3] Q. Wang, J. Wu, H. Wang, Y. Gao, Q. Liu, A. Mu, W. Ji, L. Yan, Y. Zhu, C. Zhu, X. Fang, X. Yang, Y. Huang, H. Gao, F. Liu, J. Ge, Q. Sun, X. Yang, W. Xu, Z. Liu, H. Yang, Z. Lou, B. Jiang, L. W. Guddat, P. Gong, Z. Rao, Structural basis for RNA replication by the SARS-CoV-2 polymerase. Cell 182, 417–428.e13 (2020). 32526208 10.1016/j.cell.2020.05.034 PMC7242921

[4] G. Wolff, R. W. A. L. Limpens, J. C. Zevenhoven-Dobbe, U. Laugks, S. Zheng, A. W. M. de Jong, R. I. Koning, D. A. Agard, K. Grünewald, A. J. Koster, E. J. Snijder, M. Bárcena, A molecular pore spans the double membrane of the coronavirus replication organelle. Science 369, 1395–1398 (2020). 32763915 10.1126/science.abd3629 PMC7665310

[5] B. W. Neuman, M. M. Angelini, M. J. Buchmeier, Does form meet function in the coronavirus replicative organelle? Trends Microbiol. 22, 642–647 (2014). 25037114 10.1016/j.tim.2014.06.003 PMC7127430

[6] E. J. Snijder, R. W. A. L. Limpens, A. H. de Wilde, A. W. M. de Jong, J. C. Zevenhoven-Dobbe, H. J. Maier, F. F. G. A. Faas, A. J. Koster, M. Bárcena, A unifying structural and functional model of the coronavirus replication organelle: Tracking down RNA synthesis. PLOS Biol. 18, e3000715 (2020). 32511245 10.1371/journal.pbio.3000715 PMC7302735

[7] F. Almazán, C. Galán, L. Enjuanes, The nucleoprotein is required for efficient coronavirus genome replication. J. Virol. 78, 12683–12688 (2004). 15507657 10.1128/JVI.78.22.12683-12688.2004 PMC525053

[8] R. McBride, M. van Zyl, B. C. Fielding, The coronavirus nucleocapsid is a multifunctional protein. Viruses 6, 2991–3018 (2014). 25105276 10.3390/v6082991 PMC4147684

[9] C.-H. Wu, P.-J. Chen, S.-H. Yeh, Nucleocapsid phosphorylation and RNA helicase DDX1 recruitment enables coronavirus transition from discontinuous to continuous transcription. Cell Host Microbe 16, 462–472 (2014). 25299332 10.1016/j.chom.2014.09.009 PMC7104987

[10] P. D. Burbelo, F. X. Riedo, C. Morishima, S. Rawlings, D. Smith, S. Das, J. R. Strich, D. S. Chertow, R. T. Davey, J. I. Cohen, Sensitivity in detection of antibodies to nucleocapsid and spike proteins of severe acute respiratory syndrome coronavirus 2 in patients with coronavirus disease 2019. J. Infect. Dis. 222, 206–213 (2020). 32427334 10.1093/infdis/jiaa273 PMC7313936

[11] N. K. Dutta, K. Mazumdar, J. T. Gordy, The nucleocapsid protein of SARS–CoV-2: A target for vaccine development. J. Virol. 94, e00647-20 (2020). 32546606 10.1128/JVI.00647-20 PMC7307180

[12] S. Stertz, M. Reichelt, M. Spiegel, T. Kuri, L. Martínez-Sobrido, A. García-Sastre, F. Weber, G. Kochs, The intracellular sites of early replication and budding of SARS-coronavirus. Virology 361, 304–315 (2007). 17210170 10.1016/j.virol.2006.11.027 PMC7103305

[13] M. H. Verheije, M. C. Hagemeijer, M. Ulasli, F. Reggiori, P. J. M. Rottier, P. S. Masters, C. A. M. de Haan, The coronavirus nucleocapsid protein is dynamically associated with the replication-transcription complexes. J. Virol. 84, 11575–11579 (2010). 20739524 10.1128/JVI.00569-10 PMC2953146

[14] P. V’kovski, M. Gerber, J. Kelly, S. Pfaender, N. Ebert, S. Braga Lagache, C. Simillion, J. Portmann, H. Stalder, V. Gaschen, R. Bruggmann, M. H. Stoffel, M. Heller, R. Dijkman, V. Thiel, Determination of host proteins composing the microenvironment of coronavirus replicase complexes by proximity-labeling. eLife 8, e42037 (2019). 30632963 10.7554/eLife.42037 PMC6372286

[15] K. R. Hurst, C. A. Koetzner, P. S. Masters, Characterization of a critical interaction between the coronavirus nucleocapsid protein and nonstructural protein 3 of the viral replicase-transcriptase complex. J. Virol. 87, 9159–9172 (2013). 23760243 10.1128/JVI.01275-13 PMC3754073

[16] Y. Cong, M. Ulasli, H. Schepers, M. Mauthe, P. V’kovski, F. Kriegenburg, V. Thiel, C. A. M. de Haan, F. Reggiori, Nucleocapsid protein recruitment to replication-transcription complexes plays a crucial role in coronaviral life cycle. J. Virol. 94, e01925-19 (2020). 31776274 10.1128/JVI.01925-19 PMC6997762

[17] C. Chang, M.-H. Hou, C.-F. Chang, C.-D. Hsiao, T. Huang, The SARS coronavirus nucleocapsid protein–forms and functions. Antiviral Res. 103, 39–50 (2014). 24418573 10.1016/j.antiviral.2013.12.009 PMC7113676

[18] S. Zúñiga, I. Sola, J. L. Moreno, P. Sabella, J. Plana-Durán, L. Enjuanes, Coronavirus nucleocapsid protein is an RNA chaperone. Virology 357, 215–227 (2007). 16979208 10.1016/j.virol.2006.07.046 PMC7111943

[19] H. Yao, Y. Song, Y. Chen, N. Wu, J. Xu, C. Sun, J. Zhang, T. Weng, Z. Zhang, Z. Wu, L. Cheng, D. Shi, X. Lu, J. Lei, M. Crispin, Y. Shi, L. Li, S. Li, Molecular Architecture of the SARS-CoV-2 Virus. Cell 183, 730–738.e13 (2020). 32979942 10.1016/j.cell.2020.09.018 PMC7474903

[20] S. Klein, M. Cortese, S. L. Winter, M. Wachsmuth-Melm, C. J. Neufeldt, B. Cerikan, M. L. Stanifer, S. Boulant, R. Bartenschlager, P. Chlanda, SARS-CoV-2 structure and replication characterized by in situ cryo-electron tomography. Nat. Commun. 11, 5885 (2020). 33208793 10.1038/s41467-020-19619-7 PMC7676268

[21] T.-Y. Peng, K.-R. Lee, W.-Y. Tarn, Phosphorylation of the arginine/serine dipeptide-rich motif of the severe acute respiratory syndrome coronavirus nucleocapsid protein modulates its multimerization, translation inhibitory activity and cellular localization. FEBS J. 275, 4152–4163 (2008). 18631359 10.1111/j.1742-4658.2008.06564.x PMC7164085

[22] Y.-S. Lo, S.-Y. Lin, S.-M. Wang, C.-T. Wang, Y.-L. Chiu, T.-H. Huang, M.-H. Hou, Oligomerization of the carboxyl terminal domain of the human coronavirus 229E nucleocapsid protein. FEBS Lett. 587, 120–127 (2013). 23178926 10.1016/j.febslet.2012.11.016 PMC7089611

[23] S. Kang, M. Yang, Z. Hong, L. Zhang, Z. Huang, X. Chen, S. He, Z. Zhou, Z. Zhou, Q. Chen, Y. Yan, C. Zhang, H. Shan, S. Chen, Crystal structure of SARS-CoV-2 nucleocapsid protein RNA binding domain reveals potential unique drug targeting sites. Acta Pharmaceutica Sinica B 10, 1228–1238 (2020). 32363136 10.1016/j.apsb.2020.04.009 PMC7194921

[24] Y. Peng, N. Du, Y. Lei, S. Dorje, J. Qi, T. Luo, G. F. Gao, H. Song, Structures of the SARS-CoV-2 nucleocapsid and their perspectives for drug design. EMBO J. 39, e105938 (2020). 32914439 10.15252/embj.2020105938 PMC7560215

[25] D. C. Dinesh, D. Chalupska, J. Silhan, E. Koutna, R. Nencka, V. Veverka, E. Boura, Structural basis of RNA recognition by the SARS-CoV-2 nucleocapsid phosphoprotein. PLOS Pathog. 16, e1009100 (2020). 33264373 10.1371/journal.ppat.1009100 PMC7735635

[26] Q. Huang, L. Yu, A. M. Petros, A. Gunasekera, Z. Liu, N. Xu, P. Hajduk, J. Mack, S. W. Fesik, E. T. Olejniczak, Structure of the N-terminal RNA-binding domain of the SARS CoV nucleocapsid protein. Biochemistry 43, 6059–6063 (2004). 15147189 10.1021/bi036155b

[27] J. S. Redzic, E. Lee, A. Born, A. Issaian, M. A. Henen, P. J. Nichols, A. Blue, K. C. Hansen, A. D’Alessandro, B. Vögeli, E. Z. Eisenmesser, The inherent dynamics and interaction sites of the SARS-CoV-2 nucleocapsid N-terminal region. J. Mol. Biol. 433, 167108 (2021). 34161778 10.1016/j.jmb.2021.167108 PMC8214912

[28] Q. Ye, A. M. V. West, S. Silletti, K. D. Corbett, Architecture and self-assembly of the SARS-CoV-2 nucleocapsid protein. Protein Sci. 29, 1890–1901 (2020). 32654247 10.1002/pro.3909 PMC7405475

[29] W. Zeng, G. Liu, H. Ma, D. Zhao, Y. Yang, M. Liu, A. Mohammed, C. Zhao, Y. Yang, J. Xie, C. Ding, X. Ma, J. Weng, Y. Gao, H. He, T. Jin, Biochemical characterization of SARS-CoV-2 nucleocapsid protein. Biochem. Biophys. Res. Commun. 527, 618–623 (2020). 32416961 10.1016/j.bbrc.2020.04.136 PMC7190499

[30] C. A. Lutomski, T. J. El-Baba, J. R. Bolla, C. V. Robinson, Multiple roles of SARS-CoV-2 N protein facilitated by proteoform-specific interactions with RNA, host proteins, and convalescent antibodies. JACS Au 1, 1147–1157 (2021). 34462738 10.1021/jacsau.1c00139 PMC8231660

[31] J. Cubuk, J. J. Alston, J. J. Incicco, S. Singh, M. D. Stuchell-Brereton, M. D. Ward, M. I. Zimmerman, N. Vithani, D. Griffith, J. A. Wagoner, G. R. Bowman, K. B. Hall, A. Soranno, A. S. Holehouse, The SARS-CoV-2 nucleocapsid protein is dynamic, disordered, and phase separates with RNA. Nat. Commun. 12, 1936 (2021). 33782395 10.1038/s41467-021-21953-3 PMC8007728

[32] S. Guseva, L. M. Perez, A. Camacho-Zarco, L. M. Bessa, N. Salvi, A. Malki, D. Maurin, M. Blackledge, 1H, 13C and 15N backbone chemical shift assignments of the n-terminal and central intrinsically disordered domains of SARS-CoV-2 nucleoprotein. Biomol. NMR Assign. 15, 255–260 (2021). 33730325 10.1007/s12104-021-10014-x PMC7967780

[33] M. Schiavina, L. Pontoriero, V. N. Uversky, I. C. Felli, R. Pierattelli, The highly flexible disordered regions of the SARS-CoV-2 nucleocapsid N protein within the 1-248 residue construct: Sequence-specific resonance assignments through NMR. Biomol. NMR Assign. 15, 219–227 (2021). 33660218 10.1007/s12104-021-10009-8 PMC7928198

[34] A. Savastano, A. Ibáñez de Opakua, M. Rankovic, M. Zweckstetter, Nucleocapsid protein of SARS-CoV-2 phase separates into RNA-rich polymerase-containing condensates. Nat. Commun. 11, 6041 (2020). 33247108 10.1038/s41467-020-19843-1 PMC7699647

[35] C. Iserman, C. A. Roden, M. A. Boerneke, R. S. G. Sealfon, G. A. McLaughlin, I. Jungreis, E. J. Fritch, Y. J. Hou, J. Ekena, C. A. Weidmann, C. L. Theesfeld, M. Kellis, O. G. Troyanskaya, R. S. Baric, T. P. Sheahan, K. M. Weeks, A. S. Gladfelter, Genomic RNA elements drive phase separation of the SARS-CoV-2 nucleocapsid. Mol. Cell 80, 1078–1091.e6 (2020). 33290746 10.1016/j.molcel.2020.11.041 PMC7691212

[36] H. M. Forsythe, J. Rodriguez Galvan, Z. Yu, S. Pinckney, P. Reardon, R. B. Cooley, P. Zhu, A. D. Rolland, J. S. Prell, E. Barbar, Multivalent binding of the partially disordered SARS-CoV-2 nucleocapsid phosphoprotein dimer to RNA. Biophys. J. 120, 2890–2901 (2021). 33794152 10.1016/j.bpj.2021.03.023 PMC8007181

[37] H. Chen, Y. Cui, X. Han, W. Hu, M. Sun, Y. Zhang, P.-H. Wang, G. Song, W. Chen, J. Lou, Liquid–liquid phase separation by SARS-CoV-2 nucleocapsid protein and RNA. Cell Res. 30, 1143–1145 (2020). 32901111 10.1038/s41422-020-00408-2 PMC7477871

[38] T. M. Perdikari, A. C. Murthy, V. H. Ryan, S. Watters, M. T. Naik, N. L. Fawzi, SARS-CoV-2 nucleocapsid protein phase-separates with RNA and with human hnRNPs. EMBO J. 39, e106478 (2020). 33200826 10.15252/embj.2020106478 PMC7737613

[39] C. R. Carlson, J. B. Asfaha, C. M. Ghent, C. J. Howard, N. Hartooni, M. Safari, A. D. Frankel, D. O. Morgan, Phosphoregulation of phase separation by the SARS-CoV-2 N protein suggests a biophysical basis for its dual functions. Mol. Cell 80, 1092–1103.e4 (2020). 33248025 10.1016/j.molcel.2020.11.025 PMC7677695

[40] S. Lu, Q. Ye, D. Singh, Y. Cao, J. K. Diedrich, J. R. Yates, E. Villa, D. W. Cleveland, K. D. Corbett, The SARS-CoV-2 nucleocapsid phosphoprotein forms mutually exclusive condensates with RNA and the membrane-associated M protein. Nat. Commun. 12, 502 (2021). 33479198 10.1038/s41467-020-20768-y PMC7820290

[41] A. Jack, L. S. Ferro, M. J. Trnka, E. Wehri, A. Nadgir, X. Nguyenla, D. Fox, K. Costa, S. Stanley, J. Schaletzky, A. Yildiz, SARS-CoV-2 nucleocapsid protein forms condensates with viral genomic RNA. PLOS Biol. 19, e3001425 (2021). 34634033 10.1371/journal.pbio.3001425 PMC8553124

[42] Y. Shin, C. P. Brangwynne, Liquid phase condensation in cell physiology and disease. Science 357, eaaf4382 (2017). 28935776 10.1126/science.aaf4382

[43] J. Nikolic, R. Le Bars, Z. Lama, N. Scrima, C. Lagaudrière-Gesbert, Y. Gaudin, D. Blondel, Negri bodies are viral factories with properties of liquid organelles. Nat. Commun. 8, 58 (2017). 28680096 10.1038/s41467-017-00102-9 PMC5498545

[44] S. Guseva, S. Milles, M. R. Jensen, N. Salvi, J.-P. Kleman, D. Maurin, R. W. H. Ruigrok, M. Blackledge, Measles virus nucleo- and phosphoproteins form liquid-like phase-separated compartments that promote nucleocapsid assembly. Sci. Adv. 6, eaaz7095 (2020). 32270045 10.1126/sciadv.aaz7095 PMC7112944

[45] B. W. Neuman, Bioinformatics and functional analyses of coronavirus nonstructural proteins involved in the formation of replicative organelles. Antiviral Res. 135, 97–107 (2016). 27743916 10.1016/j.antiviral.2016.10.005 PMC7113682

[46] J. Lei, Y. Kusov, R. Hilgenfeld, Nsp3 of coronaviruses: Structures and functions of a large multi-domain protein. Antiviral Res. 149, 58–74 (2018). 29128390 10.1016/j.antiviral.2017.11.001 PMC7113668

[47] B. W. Neuman, P. Chamberlain, F. Bowden, J. Joseph, Atlas of coronavirus replicase structure. Virus Res. 194, 49–66 (2014). 24355834 10.1016/j.virusres.2013.12.004 PMC7114488

[48] K. R. Hurst, R. Ye, S. J. Goebel, P. Jayaraman, P. S. Masters, An interaction between the nucleocapsid protein and a component of the replicase-transcriptase complex is crucial for the infectivity of coronavirus genomic RNA. J. Virol. 84, 10276–10288 (2010). 20660183 10.1128/JVI.01287-10 PMC2937748

[49] P. Serrano, M. A. Johnson, M. S. Almeida, R. Horst, T. Herrmann, J. S. Joseph, B. W. Neuman, V. Subramanian, K. S. Saikatendu, M. J. Buchmeier, R. C. Stevens, P. Kuhn, K. Wüthrich, Nuclear magnetic resonance structure of the n-terminal domain of nonstructural protein 3 from the severe acute respiratory syndrome coronavirus. J. Virol. 81, 12049–12060 (2007). 17728234 10.1128/JVI.00969-07 PMC2168779

[50] S. C. Keane, D. P. Giedroc, Solution structure of mouse hepatitis virus (MHV) nsp3a and determinants of the interaction with MHV nucleocapsid (N) protein. J. Virol. 87, 3502–3515 (2013). 23302895 10.1128/JVI.03112-12 PMC3592139

[51] I. Imbert, E. J. Snijder, M. Dimitrova, J.-C. Guillemot, P. Lécine, B. Canard, The SARS-Coronavirus PLnc domain of nsp3 as a replication/transcription scaffolding protein. Virus Res. 133, 136–148 (2008). 18255185 10.1016/j.virusres.2007.11.017 PMC7114086

[52] N. E. Grossoehme, L. Li, S. C. Keane, P. Liu, C. E. Dann, J. L. Leibowitz, D. P. Giedroc, Coronavirus N protein N-terminal domain (NTD) specifically binds the transcriptional regulatory sequence (TRS) and melts TRS-cTRS RNA duplexes. J. Mol. Biol. 394, 544–557 (2009). 19782089 10.1016/j.jmb.2009.09.040 PMC2783395

[53] N. Salvi, L. M. Bessa, S. Guseva, A. Camacho-Zarco, D. Maurin, L. M. Perez, A. Malki, M. Hengesbach, S. M. Korn, A. Schlundt, H. Schwalbe, M. Blackledge, 1H, 13C and 15N backbone chemical shift assignments of SARS-CoV-2 nsp3a. Biomol. NMR Assign. 15, 173–176 (2021). 33475934 10.1007/s12104-020-10001-8 PMC7819138

[54] D. Oudshoorn, K. Rijs, R. W. A. L. Limpens, K. Groen, A. J. Koster, E. J. Snijder, M. Kikkert, M. Bárcena, Expression and cleavage of middle east respiratory syndrome coronavirus nsp3-4 polyprotein induce the formation of double-membrane vesicles that mimic those associated with coronaviral RNA replication. MBio 8, e01658-17 (2017). 29162711 10.1128/mBio.01658-17 PMC5698553

[55] S. M. Korn, R. Lambertz, B. Fürtig, M. Hengesbach, F. Löhr, C. Richter, H. Schwalbe, J. E. Weigand, J. Wöhnert, A. Schlundt, 1H, 13C, and 15N backbone chemical shift assignments of the C-terminal dimerization domain of SARS-CoV-2 nucleocapsid protein. Biomol. NMR Assign. 15, 129–135 (2021). 33270159 10.1007/s12104-020-09995-y PMC7711055

[56] J. Song, L. K. Durrin, T. A. Wilkinson, T. G. Krontiris, Y. Chen, Identification of a SUMO-binding motif that recognizes SUMO-modified proteins. Proc. Natl. Acad. Sci. U.S.A. 101, 14373–14378 (2004). 15388847 10.1073/pnas.0403498101 PMC521952

[57] L. Zinzula, J. Basquin, S. Bohn, F. Beck, S. Klumpe, G. Pfeifer, I. Nagy, A. Bracher, F. U. Hartl, W. Baumeister, High-resolution structure and biophysical characterization of the nucleocapsid phosphoprotein dimerization domain from the Covid-19 severe acute respiratory syndrome coronavirus 2. Biochem. Biophys. Res. Commun. 538, 54–62 (2021). 33039147 10.1016/j.bbrc.2020.09.131 PMC7532810

[58] C.-K. Chang, Y.-L. Hsu, Y.-H. Chang, F.-A. Chao, M.-C. Wu, Y.-S. Huang, C.-K. Hu, T.-H. Huang, Multiple nucleic acid binding sites and intrinsic disorder of severe acute respiratory syndrome coronavirus nucleocapsid protein: Implications for ribonucleocapsid protein packaging. J. Virol. 83, 2255–2264 (2009). 19052082 10.1128/JVI.02001-08 PMC2643731

[59] S. Guseva, S. Milles, M. R. Jensen, G. Schoehn, R. W. Ruigrok, M. Blackledge, Structure, dynamics and phase separation of measles virus RNA replication machinery. Curr. Opin. Virol. 41, 59–67 (2020). 32570195 10.1016/j.coviro.2020.05.006

[60] K. H. Gardner, L. E. Kay, The use of ^2^H, ^13^C, ^15^N multidimensional NMR GTO study the structure and dynamics of proteins. Annu. Rev. Biophys. Biomol. Struct. 27, 357–406 (1998). 9646872 10.1146/annurev.biophys.27.1.357

[61] F. Delaglio, S. Grzesiek, G. Vuister, G. Zhu, J. Pfeifer, A. Bax, NMRPipe: A multidimensional spectral processing system based on UNIX pipes. J. Biomol. NMR 6, 277–293 (1995). 8520220 10.1007/BF00197809

[62] W. Lee, M. Tonelli, J. L. Markley, NMRFAM-SPARKY: Enhanced software for biomolecular NMR spectroscopy. Bioinformatics 31, 1325–1327 (2015). 25505092 10.1093/bioinformatics/btu830 PMC4393527

[63] N.-A. Lakomek, J. Ying, A. Bax, Measurement of ^15^N relaxation rates in perdeuterated proteins by TROSY-based methods. J. Biomol. NMR 53, 209–221 (2012). 22689066 10.1007/s10858-012-9626-5 PMC3412688

[64] D. Marion, P. C. Driscoll, L. E. Kay, P. T. Wingfield, A. Bax, A. M. Gronenborn, G. M. Clore, Overcoming the overlap problem in the assignment of 1H NMR spectra of larger proteins by use of three-dimensional heteronuclear 1H-15N Hartmann-Hahn-multiple quantum coherence and nuclear Overhauser-multiple quantum coherence spectroscopy: Application to interleukin 1 beta. Biochemistry 28, 6150–6156 (1989). 2675964 10.1021/bi00441a004

[65] R. T. Clowes, W. Boucher, C. H. Hardman, P. J. Domaille, E. D. Laue, A 4D HCC(CO)NNH experiment for the correlation of aliphatic side-chain and backbone resonances in 13C/15N-labelled proteins. J. Biomol. NMR 3, 349–354 (1993).

[66] L. E. Kay, G. Y. Xu, A. U. Singer, D. R. Muhandiram, J. D. Formankay, A Gradient-Enhanced HCCH-TOCSY experiment for recording side-chain ^1^H and ^13^C Correlations in H_2_O samples of proteins. J. Magn. Reson. 101, 333–337 (1993).

[67] E. Lescop, P. Schanda, B. Brutscher, A set of BEST triple-resonance experiments for time-optimized protein resonance assignment. J. Magn. Reson. 187, 163–169 (2007). 17468025 10.1016/j.jmr.2007.04.002

[68] J. A. Marsh, V. K. Singh, Z. Jia, J. D. Forman-Kay, Sensitivity of secondary structure propensities to sequence differences between alpha- and gamma-synuclein: Implications for fibrillation. Protein Sci. 15, 2795–2804 (2006). 17088319 10.1110/ps.062465306 PMC2242444

[69] R. R. Ernst, G. Bodenhausen, A. Wokaun, *Principles of Nuclear Magnetic Resonance in One and Two Dimensions* (Oxford Univ. Press, 1988).

[70] P. Pernot, A. Round, R. Barrett, A. De Maria Antolinos, A. Gobbo, E. Gordon, J. Huet, J. Kieffer, M. Lentini, M. Mattenet, C. Morawe, C. Mueller-Dieckmann, S. Ohlsson, W. Schmid, J. Surr, P. Theveneau, L. Zerrad, S. McSweeney, Upgraded ESRF BM29 beamline for SAXS on macromolecules in solution. J. Synchrotron Radiat. 20, 660–664 (2013). 23765312 10.1107/S0909049513010431 PMC3943554

[71] A. J. Nederveen, J. F. Doreleijers, W. Vranken, Z. Miller, C. A. E. M. Spronk, S. B. Nabuurs, P. Güntert, M. Livny, J. L. Markley, M. Nilges, E. L. Ulrich, R. Kaptein, A. M. J. J. Bonvin, RECOORD: A recalculated coordinate database of 500+ proteins from the PDB using restraints from the BioMagResBank. Proteins 59, 662–672 (2005). 15822098 10.1002/prot.20408

[72] V. Ozenne, F. Bauer, L. Salmon, J.-R. Huang, M. R. Jensen, S. Segard, P. Bernadó, C. Charavay, M. Blackledge, Flexible-meccano: A tool for the generation of explicit ensemble descriptions of intrinsically disordered proteins and their associated experimental observables. Bioinformatics 28, 1463–1470 (2012). 22613562 10.1093/bioinformatics/bts172

[73] D. Franke, M. V. Petoukhov, P. V. Konarev, A. Panjkovich, A. Tuukkanen, H. D. T. Mertens, A. G. Kikhney, N. R. Hajizadeh, J. M. Franklin, C. M. Jeffries, D. I. Svergun, *ATSAS 2.8*: A comprehensive data analysis suite for small-angle scattering from macromolecular solutions. J. Appl. Cryst. 50, 1212–1225 (2017). 28808438 10.1107/S1600576717007786 PMC5541357

[74] L. Salmon, G. Nodet, V. Ozenne, G. Yin, M. R. Jensen, M. Zweckstetter, M. Blackledge, NMR characterization of long-range order in intrinsically disordered proteins. J. Am. Chem. Soc. 132, 8407–8418 (2010). 20499903 10.1021/ja101645g

