# Supplementary material for: The intrinsically disordered SARS-CoV-2 nucleoprotein in dynamic complex with its viral partner nsp3a
Source: Sci Adv. 2022 Jan 19;8(3):eabm4034. doi: 10.1126/sciadv.abm4034 (PMC8769549; doi:10.1126/sciadv.abm4034)
Supplement: Supplementary file 1 — Figs. S1 to S12 Tables S1 to S3 [file sciadv.abm4034_sm.pdf]

Science Advances
AAAS

Supplementary Materials for

The intrinsically disordered SARS-CoV-2 nucleoprotein in dynamic complex with its viral partner nsp3a

Luiza Mamigonian Bessa, Serafima Guseva, Aldo R. Camacho-Zarco, Nicola Salvi, Damien Maurin, Laura Mariño Perez, Maiia Botova, Anas Malki, Max Nanao, Malene Ringkjøbing Jensen, Rob W. H. Ruigrok, Martin Blackledge*

*Corresponding author. Email: martin.blackledge@ibs.fr

Published 19 January 2022, *Sci. Adv.* **8**, eabm4034 (2022)
DOI: 10.1126/sciadv.abm4034

This PDF file includes:

Figs. S1 to S12
Tables S1 to S3

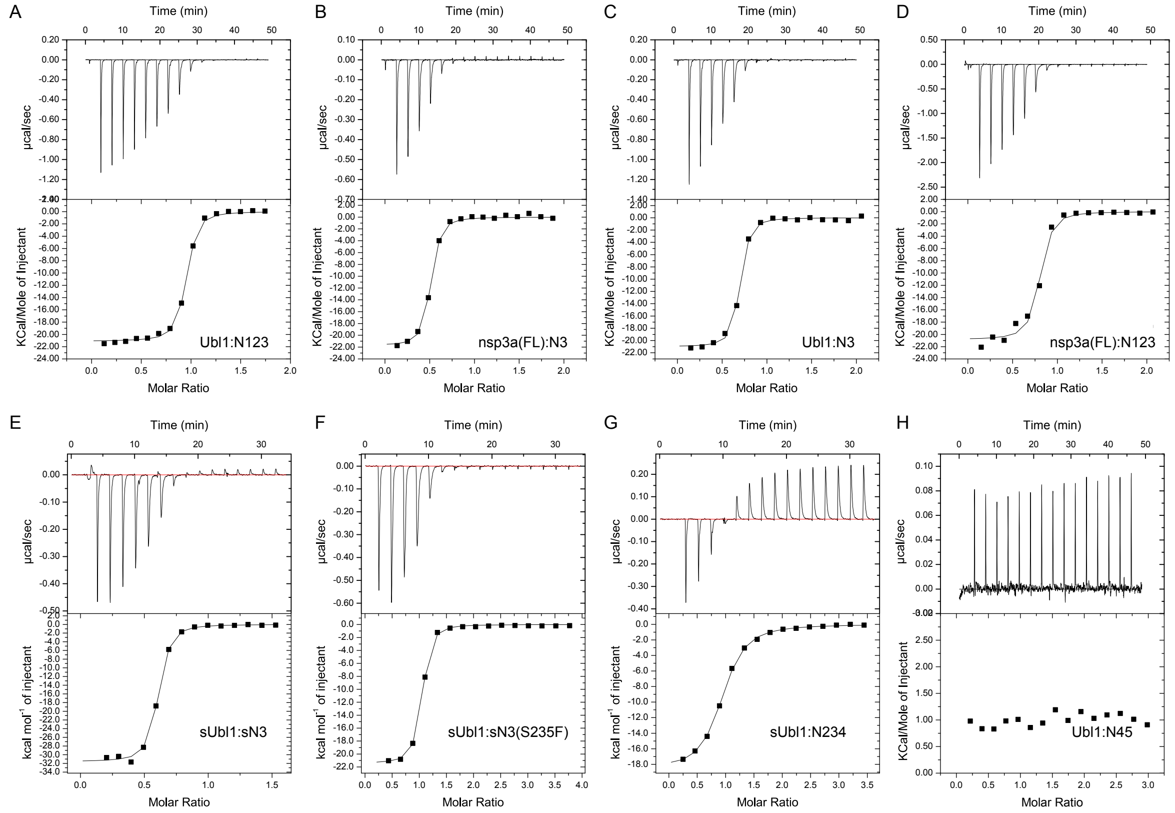

Supporting figure S1

Isothermal titration calorimetry (ITC) of different constructs of ns3pa and N. All titrations were performed at 298K, adding aliquots of N (sN3, N3, N45, N234 and N123) to either Ubl1 (1-111), sUbl1 (16-111) or Nsp3a (1-206). All data were fitted to a two-state model. Parameters are listed below in table S1.

Table S1. Thermodynamic parameters fitted to experimental ITC curves.

	K_D(nM)	ΔH(kcal.mol^{-1})	ΔS (cal.M^{-1}deg^{-1})	N
N123:Nsp3a	131±25	21.6±4.0	-41	0.665±0.007
N3:Nsp3a	82±15	21.8±2.0	-41	0.462±0.003
N123:Ubl1	72±12	21.1±1.0	-38	0.903±0.003
N3:Ubl1	70±10	19.6±1.5	-33	0.86±0.003
N45:Nsp3a	N/A	N/A	N/A	N/A
sN3:sUbl1	30±10	31.6±0.3	-71.5	0.567±0.003
sN3(S235F):sUbl1	57±8	21.4±0.3	-39	0.95±0.003
N234:sUbl1	470±20	18.7±1.6	-34	0.89±0.005

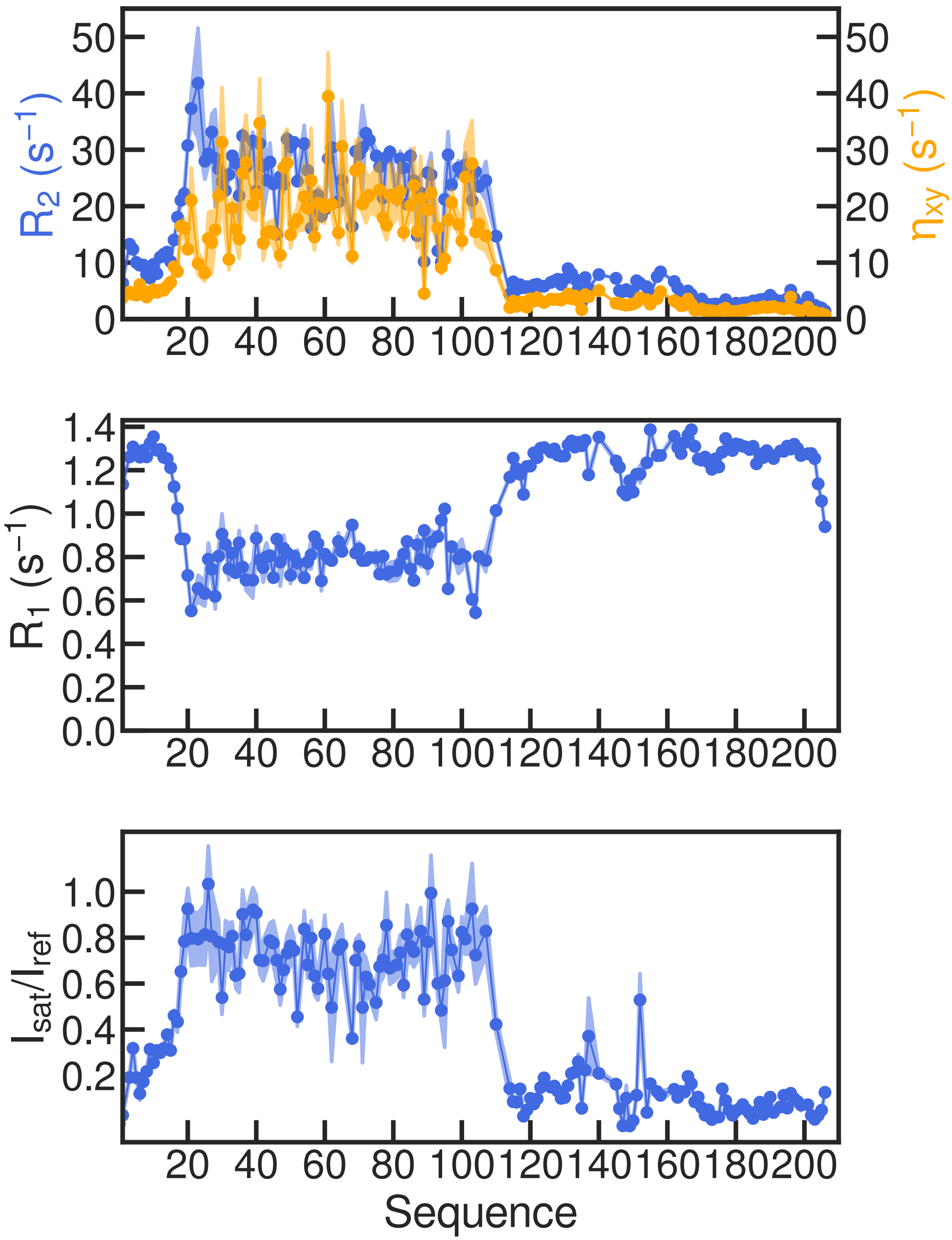

Supporting figure S2

Dynamic behaviour of the isolated two-domain nsp3a protein. ^{15}N NMR relaxation ($R_{1\rho}$, R_1, η_{xy} and heteronuclear nOe) were measured at 850MHz and 298K. Protein concentration was 800μM. Two flexible domains are immediately recognizable by their distinct relaxation properties.

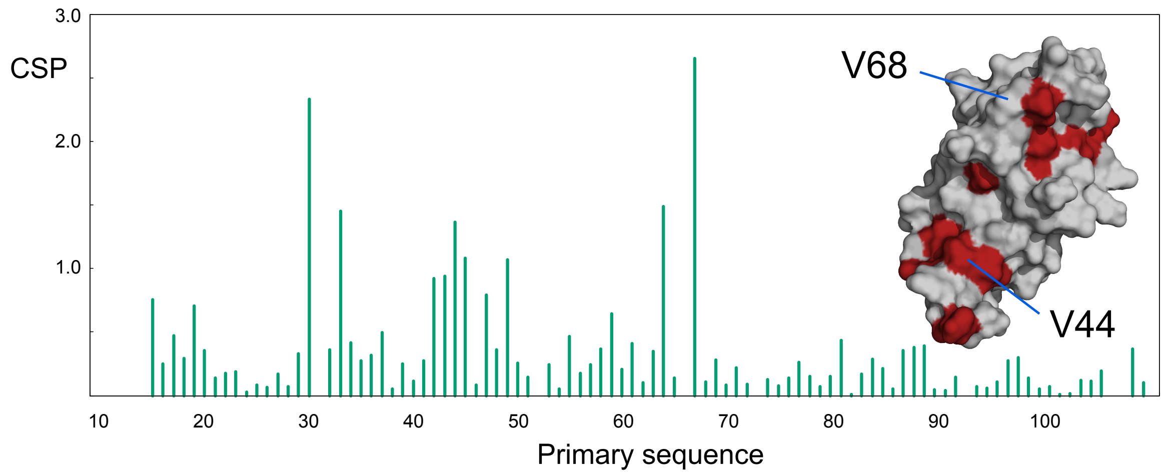

Supporting figure S3

CSP of interaction surface on Ubl1. Combined ^{15}N-^{1}H chemical shift perturbations (CSPs) mapped along the primary sequence of sUbl1 in complex with sN3 (derived from the spectrum shown in figure 2 in the main manuscript) measured on a 1:1 mixture at 200μM concentration of protein in NMR buffer. Inset - Mapping of values greater than 0.5ppm onto the surface of the NMR structure sUbl1 in complex with sN3 as determined in this manuscript.

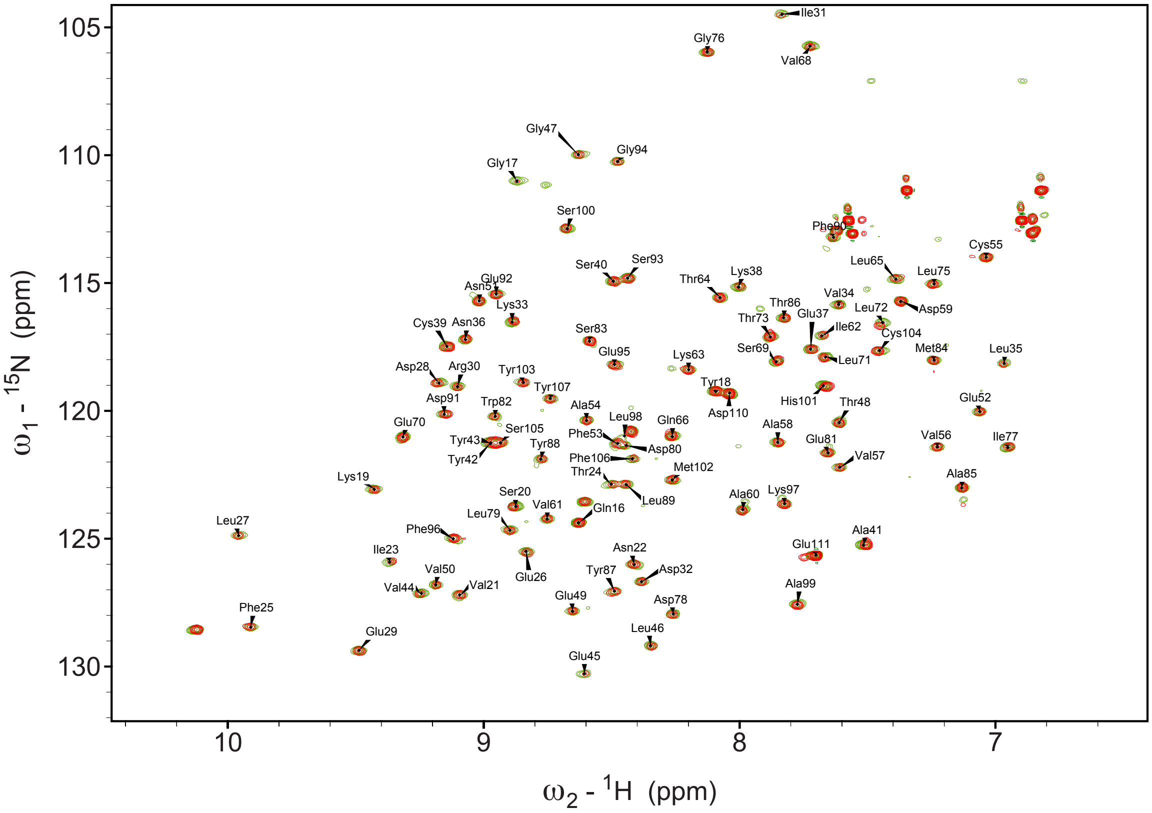

Supporting figure S4

Comparison of S235F and original sN3 on interaction with sUbl1. ^{15}N-^{1}H HSQC of sUbl1 in 1:1 complex with sN3 (spectrum shown in figure 2 in the main manuscript, green) compared to sUbl1 in 1:1 complex with the S235F mutant (alpha variant - shown in red). This comparison supports the conclusion derived from ITC (figure S1, table S1) showing that the complex is not significantly affected by this mutation. Spectra recorded at 298K, 850MHz and 200µM.

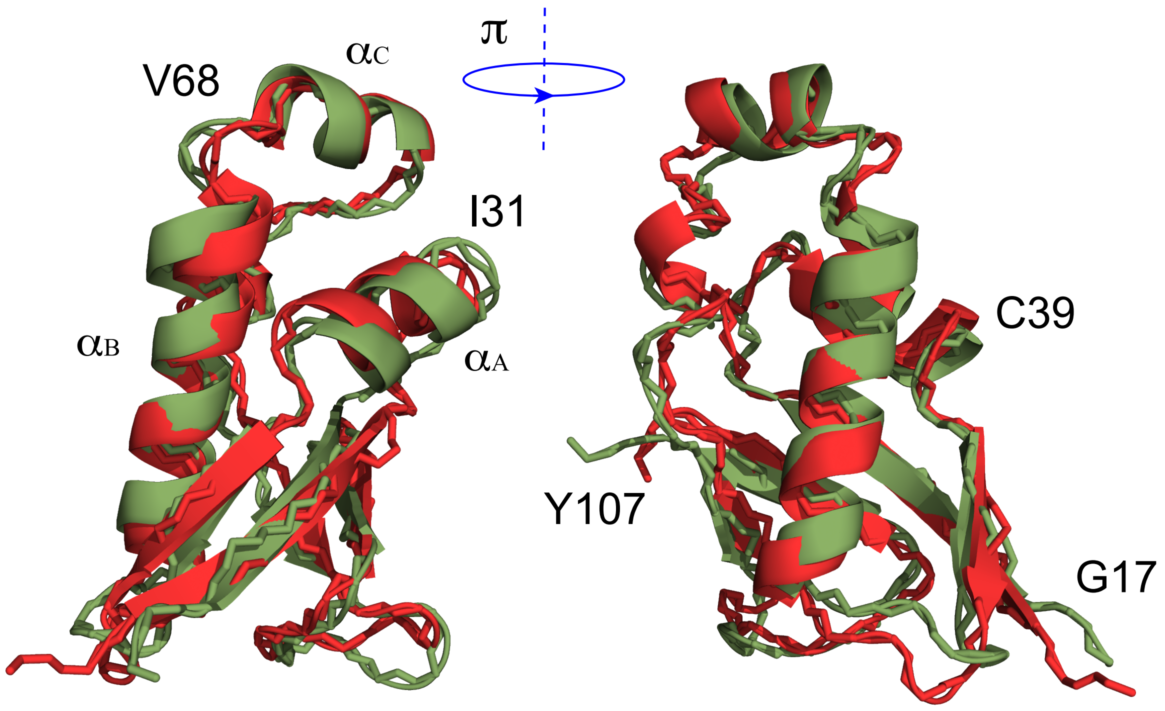

Supporting figure S5

Comparison of bound and free forms of sUbl1. X-ray crystallographic coordinates (red) were taken from the recently deposited structure from Stogios et al (pdb code 7kag). NMR structure of sUbl1 (green) in complex is the lowest restraint energy structure present in the NMR bundle determined here. Key differences are localized at Leu65 (at the C-terminus of helix α_B), Cys39 (C-terminus of helix α_A) in the main α_1 N3 binding site.

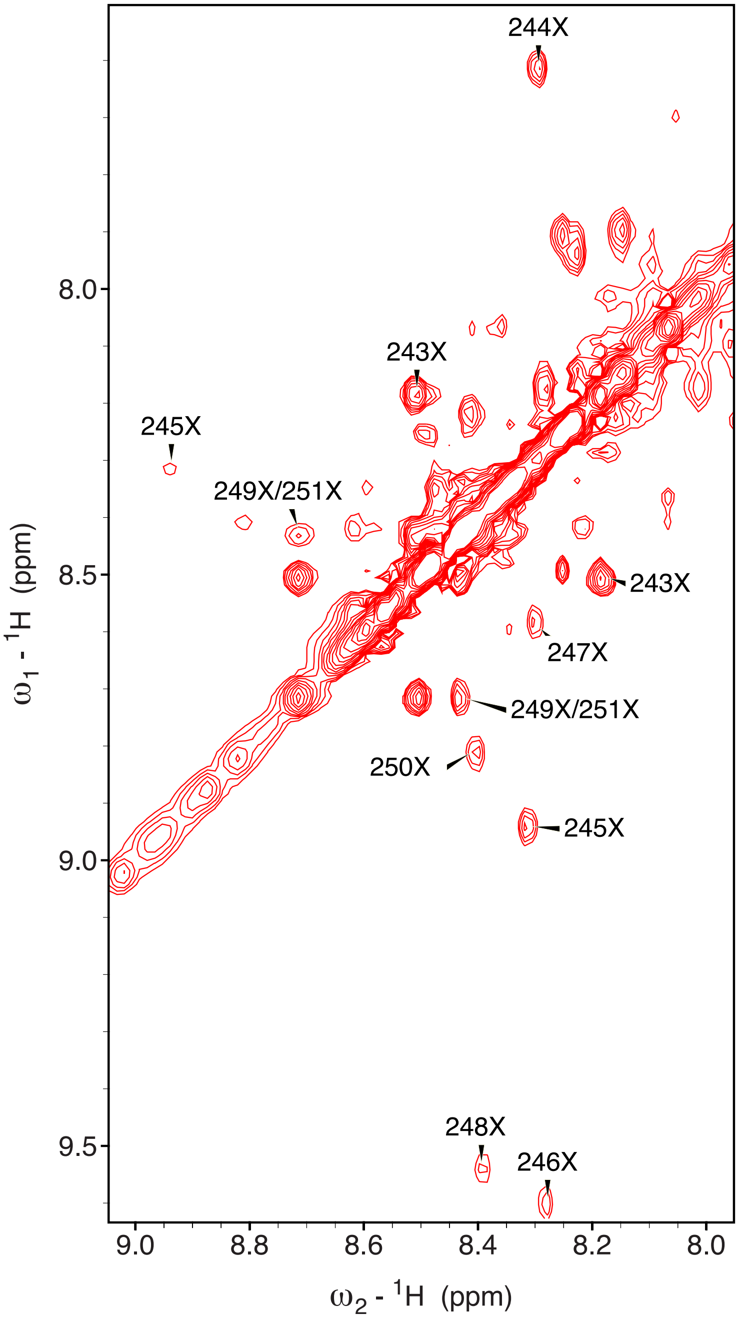

Supporting figure S6

EXSY exchange cross peaks in $\beta\alpha_2$ binding site. Exchange cross-peaks identified in the ^{1}H-^{1}H NOESY (EXSY) measured on a 1:1 complex of sUbl1 and sN3 at 600MHz and 298K. Mixing times were measured between 10 and 700ms (75ms is shown in the figure). A continuous stretch of 9 amino acids, from G243 to A251 exhibit exchange cross peaks reporting on binding and unbinding of the $\beta\alpha_2$ site.

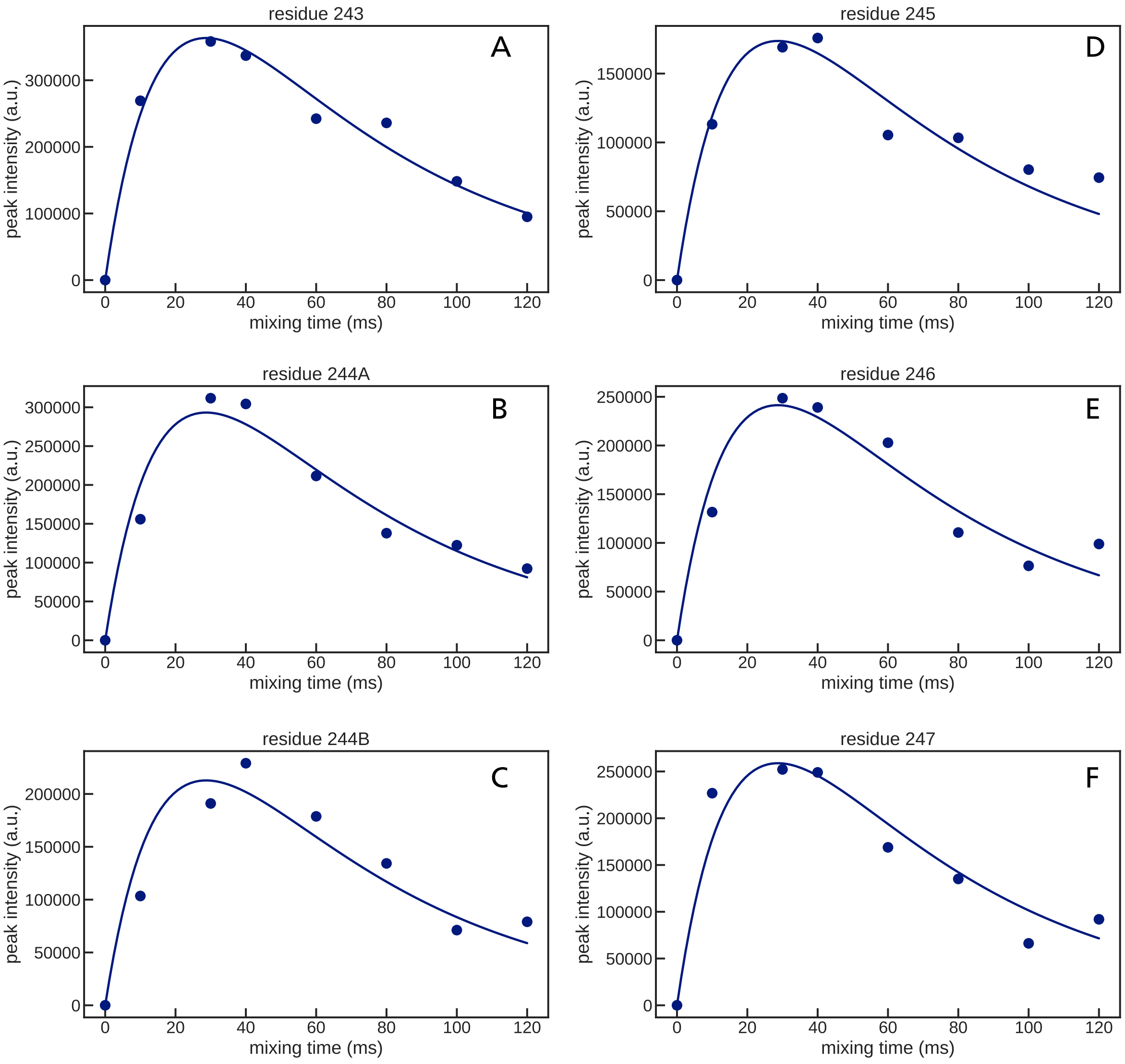

Supporting figure S7

Characterisation of exchange between free and bound forms of the $\beta\alpha_2$ site.

A-F - Examples of the 9 build-up curves fitted to evolution of EXSY cross-peak intensities using expressions describing the evolution of longitudinal exchange phenomena. Experiments were performed on a 1:1 mixture of sUbl1:sN3 at a concentration of 300μM at 600MHz. The data were fitted simultaneously as described in the Methods, resulting in an estimated common apparent exchange rate of 42 ± 13 s^{-1} (and a ^{1}H R_1 = 18 ± 3 s-1).

G - Apparent k_{ex} ($k_{ex,app}$) as a function of k_{ab}, using equation 9.8.2 in reference (69), assuming R_{1A}=R_{1B} and a true k_{ex} = 42 s^{-1}. This shows that $k_{ex,app}$ is always smaller than the true value, and $k_{ex} = k_{ex,app}$ if p_a=p_b.

H - Dependence of $k_{ex,app}$ on k_{ab} for different values of p_a. Assuming a population of the complex > 0.9, $k_{ex,app}$ = 42 s^{-1} implies that k_{ab} > 2 s^{-1} (value extracted assuming p_a =0.99) and < 7 s^{-1} (value extracted if p_a =0.9). From this, we can conclude that 63 < k_{ba} < 198 s^{-1} and 70 < k_{ex} < 200 s^{-1}.

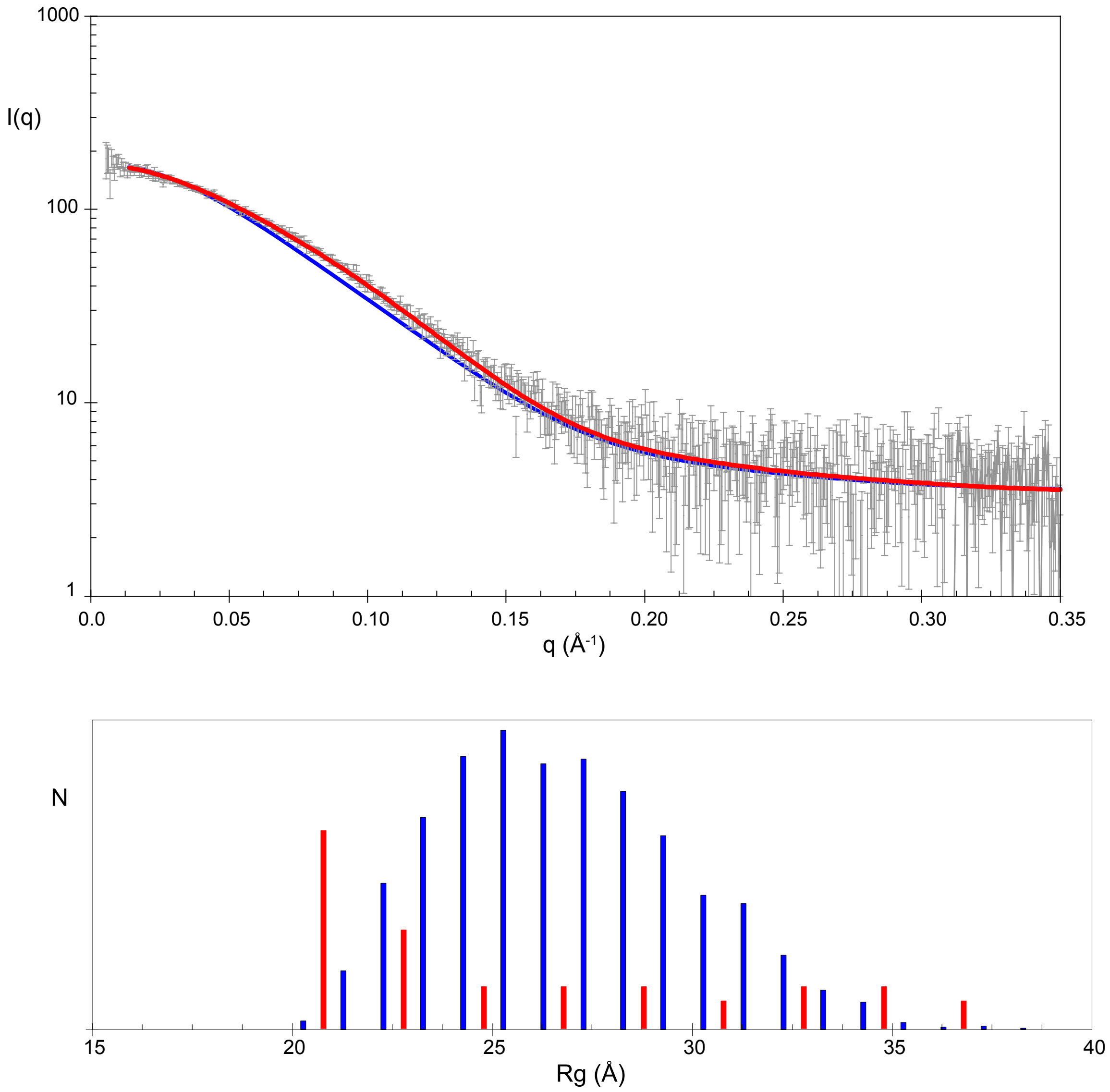

Supporting figure S8

Small angle X-ray scattering reveals a compact sUbl1:sN3 complex.

Top – Experimental (grey) and simulated (red) SAXS data from the Ubl1:N3 1:1 complex at a concentration of 300μM and temperature of 298K. Simulated data resulted from ensembles of conformations selected from a pool of 10000 structures of the Ubl1:N3 complex (see Methods). The red line represents an ensemble of 7 structures (no difference in averages radius of gyration was observed when ensembles of 5-10 conformers were used), selected using the algorithm ASTEROIDS (*74*). The blue line shows the average over the full ensemble. Measurements were acquired at the European Synchrotron Research Facility (ESRF) in Grenoble France on beamline BM29.

Bottom – Distribution of radii of gyration for the full ensemble of 10000 structures (blue) and 5 ensembles of 7 structures (red) selected using ASTEROIDS, showing that more compact structures are required to reproduce the experimental data.

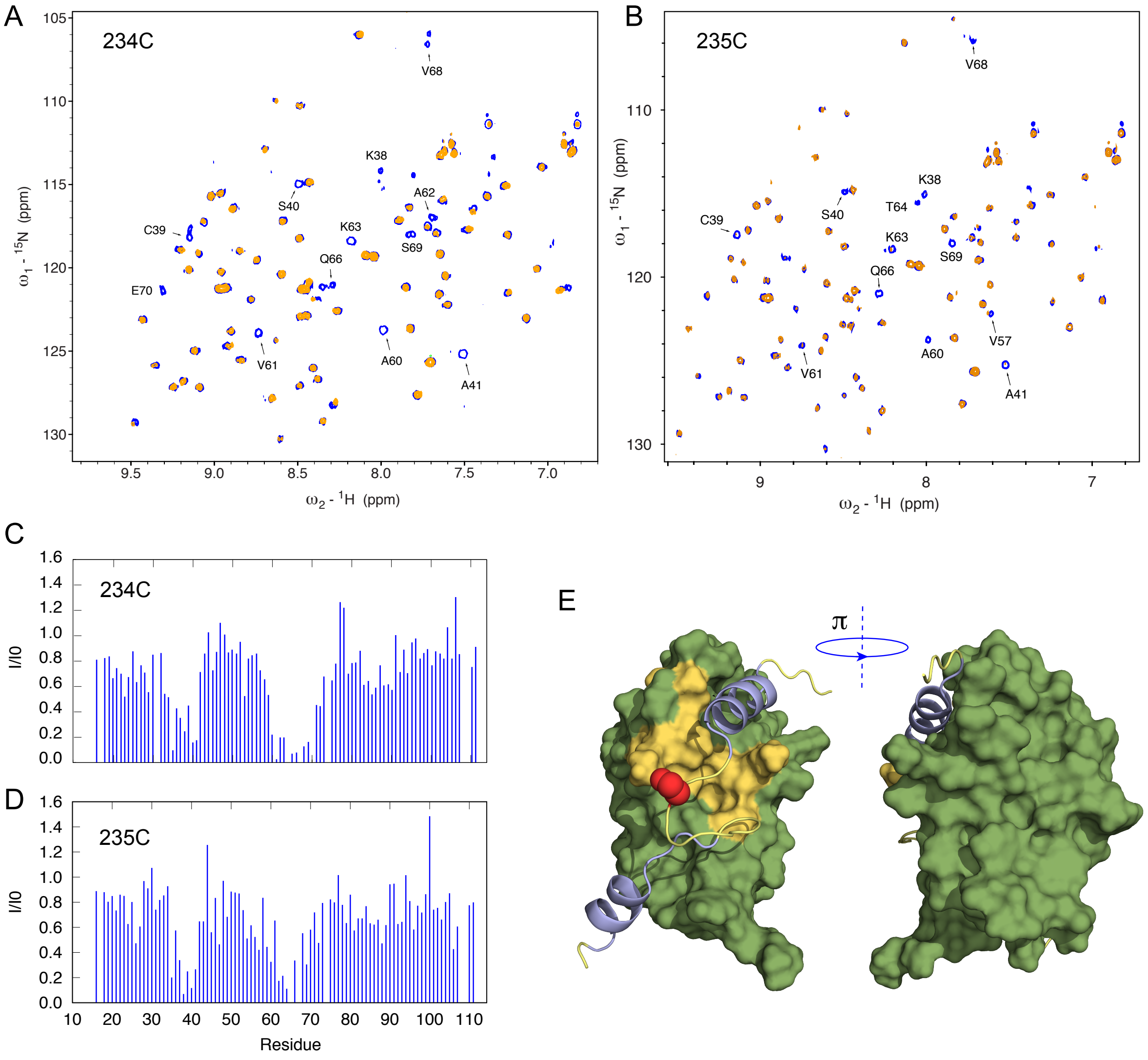

Supporting figure S9

Paramagnetic relaxation enhancement validates the NOESY derived structure. A – ^{15}N-^{1}H HSQC of sUbl1 in 1:1 complex (3400µM) with M234C mutant of sN3. Spectra recorded at 850MHz in the presence of oxidised and reduced forms of TEMPO-maleimide present on cysteine 234. B – ^{15}N-^{1}H HSQC of sUbl1 in 1:1 complex with S235C mutant of sN3. Spectra recorded at 850MHz in presence of oxidised and reduced forms of TEMPO-maleimide present on cysteine 235. C, D - Paramagnetic relaxation enhancements (PREs), derived from intensity ratios comparing spectra in A and in B respectively. E – representation of regions showing broadening on Ubl1 in the presence of spin labelled C235. Orange surface represents region with PREs lower than 0.5. Position of C235 in the flexible loop is shown in red.

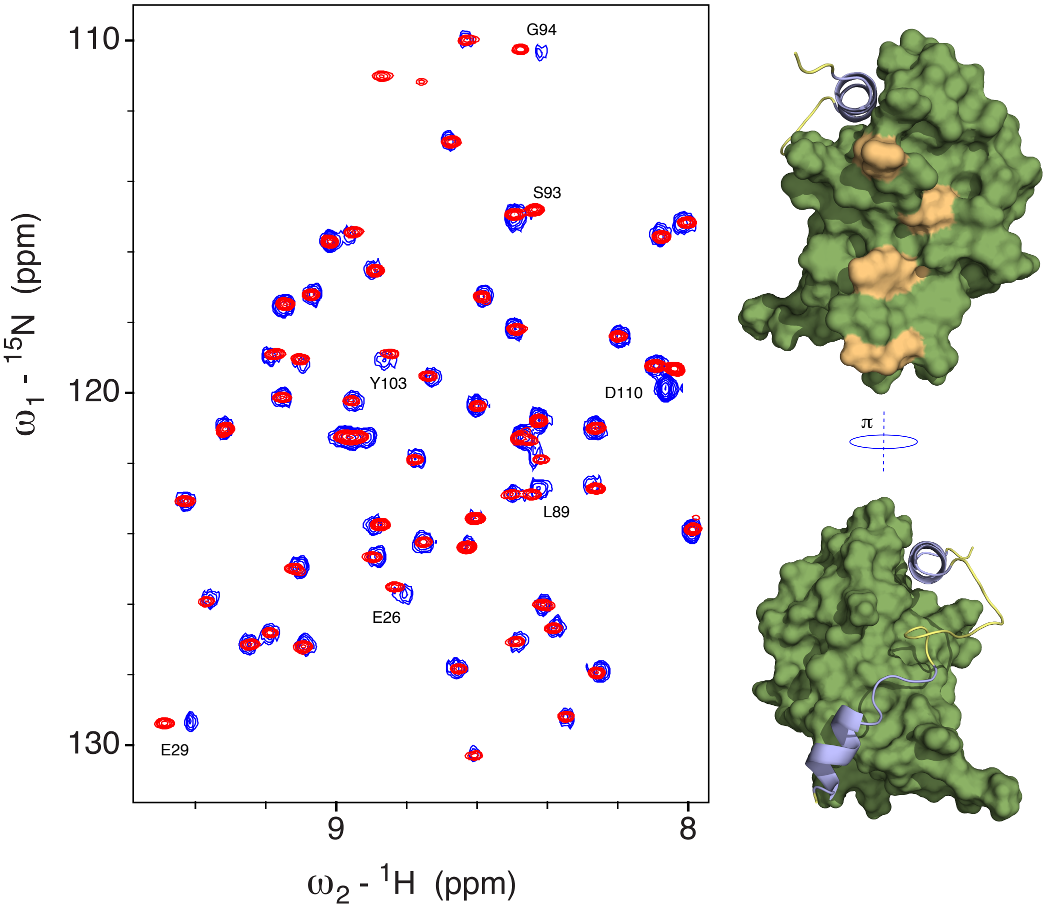

Supporting figure S10

Evidence for transient contacts between sN3 (191-213) and Ubl1. Excerpt from the ^{15}N-^{1}H HSQC of sUbl1 in 1:1 complex with sN3 (spectrum shown in red) compared to sUbl1 in 1:1 complex with a truncated form of sN3 (215-263, shown in blue). The small CSPs induced in the sUbl1 spectrum are all located on the opposing surface of Ubl1 to the main interaction site, as expected if transient weak interactions of the N-terminal region of sN3 (191-213) were no longer present, again validating the sense of the α_1 binding site. Spectra recorded at 298K, 850MHz and 300μM.

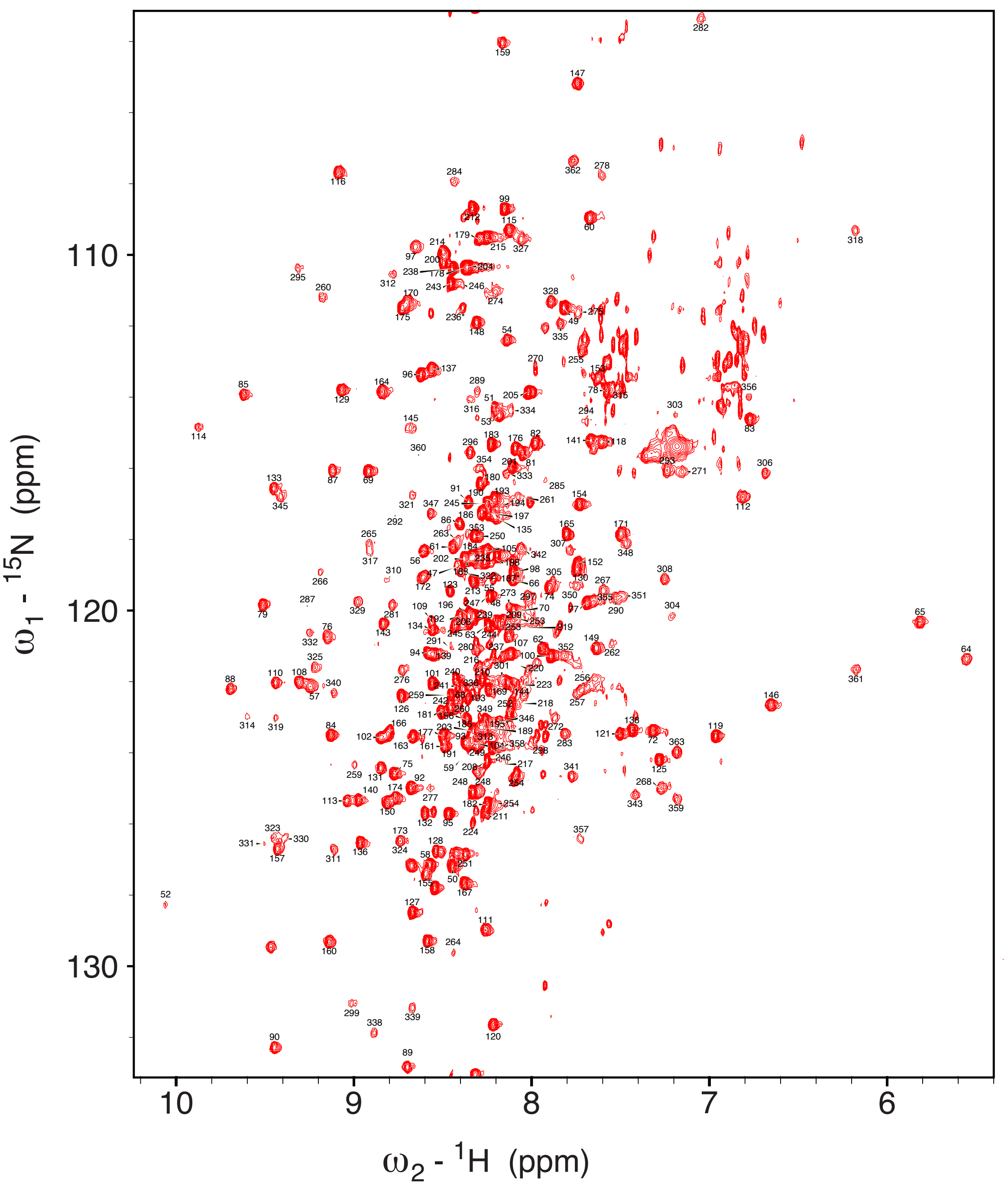

Supporting figure S11

Assigned ^{15}N-^{1}H HSQC of N234 (850MHz, 298K, 380µM). Assignments from regions corresponding to N2 and N4 were taken directly from recent publications (*25*, *55*), assignment of N3 was taken from our recent publication (*44*). Negligible CSPs were observed between published assignments for the isolated domains and the three domains in tandem, demonstrating structural independence of N2 and N4 in the isolated form of the protein.

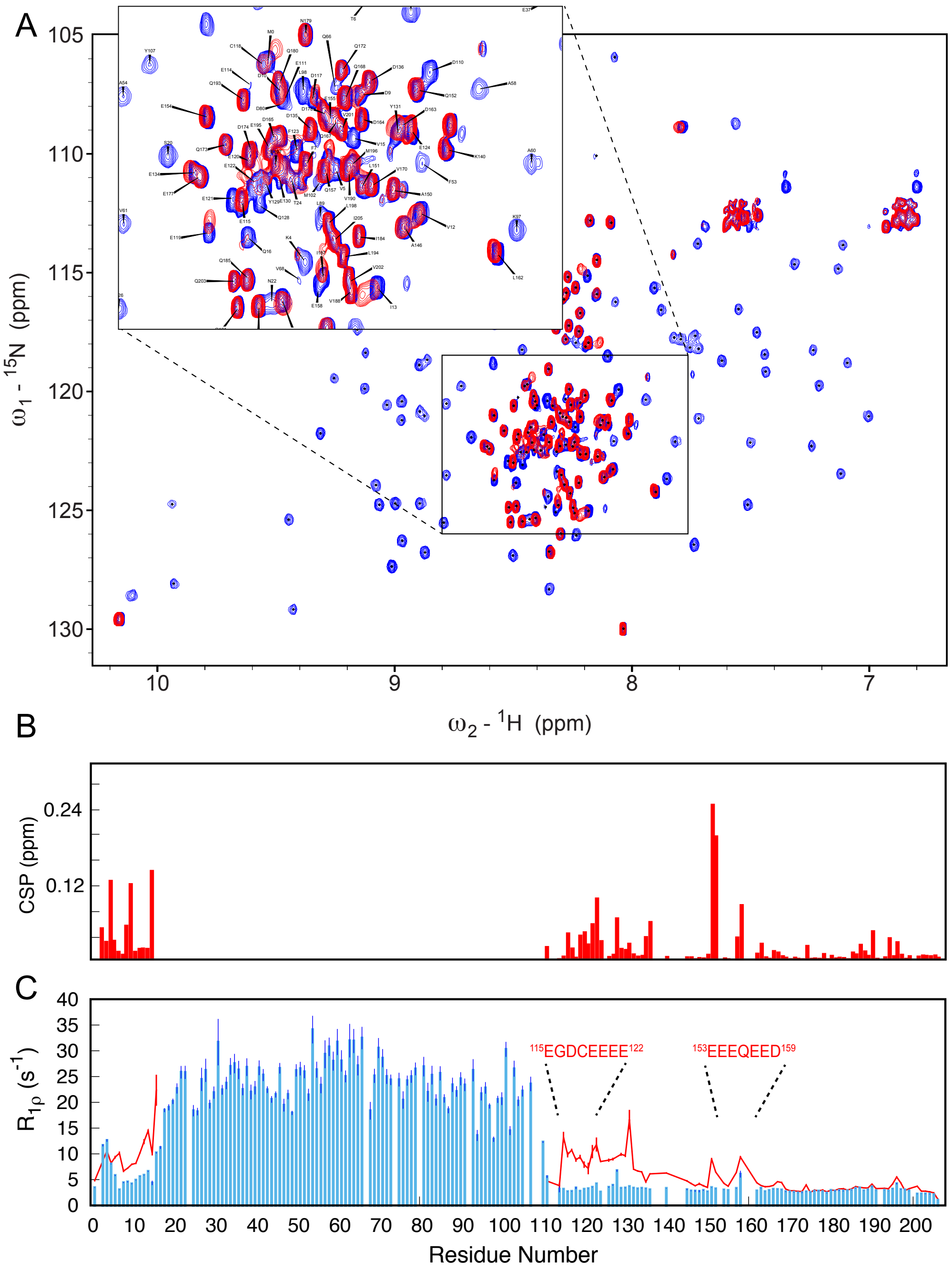

Supporting figure S12

Transient interactions between disordered tail of nsp3a and N234:Ubl1. The disordered domain of nsp3a interacts transiently with N234 in complex with Ubl1 via basic strands distributed along the chain. A - ^{15}N relaxation ($R_{1\rho}$) measured at 298K, 850MHz and 200μM of free (blue) ^{2}D labelled nsp3a, and ^{2}D labelled nsp3a in 1:1 complex with Ubl1. B, C – CSPs and assigned ^{15}N-^{1}H HSQC of the two samples (850MHz, 298K, 200μM), showing chemical shifts located in the N-terminal region and in the negatively charged 110-130 region of nsp3a.

Table S2

NMR structure determination statistics

Distance constraints	
Total NOE	597
Intermolecular	54
Intramolecular	543
Intraresidue	71
Sequential	126
Medium range (i=j±2,3,4)	87
Long range (|i-j|>4)	240
Ambiguous	64
Hydrogen bonds	39
Dihedral angle restraints	
Phi	66
Psi	66
Structural statistics [a]	
Violations (mean±s.d.)	
Distance constraints (>0.2Å)	0.03±0.03
Dihedral angle constraints	0.0±0.0
Maximum distance violation	0.16
Maximum dihedral angle violation	2.02
Deviations from idealized geometry	
Bonds (Å)	0.006±0.0001
Angles (º)	0.62±0.02
Improper dihedrals (º)	0.44±0.02
Percentage residues in favoured and allowed regions of Ramachandran plot	97.1
Average pairwise rms deviation (Å)	
Heavy (Ubl1)	2.36±0.30
Backbone (Ubl1)	1.41±0.26
Heavy (sN3)	2.65±0.54
Backbone (sN3)	1.80±0.42
Heavy (sN3:Ubl1)	2.50±0.30
Backbone (sN3:Ubl1)	1.60±0.26
Backbone wrt medoid (sN3:Ubl1)	0.70±0.12

a – The structured part of the complex is taken into consideration: Ubl1 (16-110) and N3 (218-231, 244-257).

Table S3
SAS data acquisition, sample details, data analysis, modelling fitting and software used.

Sample	N3:Ubl1	N234:Ubl1	N234
Organism	SARS Cov 2	SARS Cov 2	SARS Cov 2
Source	*E. coli* BL21(DE3)/BL21(A1)	*E. coli* BL21(DE3)/BL21(A1)	*E. coli* BL21(DE3)/BL21(A1)
Sequence	NC_045512.2	NC_045512.2	NC_045512.2
Ext Coeff (e) $M^{-1}cm^{-1}$	14565	43890	43890
Mass (Da)	21674.17	43836.77	34622.61
Concentration	0.5, 1.0, 2.0 mg/ml	0.5, 1.0 mg/ml	0.5, 1.0 mg/ml
Solvent	50 mM Na-Phosphate pH 6.5, 250 mM NaCl 2mM DTT buffer	50 mM Na-Phosphate pH 6.5, 250 mM NaCl 2mM DTT buffer	50 mM Na-Phosphate pH 6.5, 250 mM NaCl 2mM DTT buffer
Source, beamline	ESR BM29 BioSAXS	ESR BM29 BioSAXS	ESR BM29 BioSAXS
Wavelength	0.99Å	0.99Å	0.99Å
Detector model	Pilatus 2M	Pilatus 2M	Pilatus 2M
Detector type	CMOS hybrid pixel	CMOS hybrid pixel	CMOS hybrid pixel
Beam geometry (size,sample-to-detector distance)	0.7 mm x 0.7 mm, 2.867 m	0.7 mm x 0.7 mm, 2.867 m	0.7 mm x 0.7 mm, 2.867 m
q-measurement range ($Å^{-1}$)	0.0039 – 0.49	0.0039 – 0.49	0.0039 – 0.49
Energy range (keV)	12.5	12.5	12.5
Absolute scaling method	Comparison with buffer scatterring	Comparison with buffer scatterring	Comparison with buffer scatterring
Basis for normalization to constant counts	Transmitted beam intensity measured by integrated beamstop diode (*70*)	Transmitted beam intensity measured by integrated beamstop diode (*70*)	Transmitted beam intensity measured by integrated beamstop diode (70)
Method for monitoring radiation damage, X-ray dose where relevant	Inspection of each frame	Inspection of each frame	Inspection of each frame
Exposure time, number of exposures	1s/frame, 10 frames	1s/frame, 10 frames	1s/frame, 10 frames
Sample temperature	298K	298K	298K
SAS data reduction to sample–solvent scattering	Solvent substraction, averaging using BM29 online processing	Solvent substraction, averaging using BM29 online processing	Solvent substraction, averaging using BM29 online processing
Analytical method	Comparison of SAXS curves predicted from an ensemble of conformations of N3:Ubl1 complex plus statistical coil sampling (72)	Comparison of SAXS curves predicted from individual conformations of N234/Ubl1 complex plus statistical coil sampling (72)	Comparison of SAXS curves predicted from an ensemble of conformations of N234 generated using statistical coil sampling (72)
SAXS curve prediction software	Crysol (73)	Crysol3 (73)	Crysol (73)
Fitting software	ASTEROIDS (74) ensemble selection	Crysol3 (73) Default settings	No fitting
$\chi 2$	1.08	1.36	N/A
Predicted Rg (Å)	24.9	37.1	N/A
Vol ($Å^3$), Ra (Å), Dro ($eÅ^{-3}$)	N/A	122041	N/A
Guinier analysis range ($Å^{-1}$)	0.01-0.03	0.01-0.03	N/A
Simulation/data comparison range ($Å^{-1}$)	0.01-0.35	0.01-0.35	0.01-0.35
